# A Novel 167‐Amino Acid Protein Encoded by CircPCSK6 Inhibits Intrahepatic Cholangiocarcinoma Progression via IKBα Ubiquitination

**DOI:** 10.1002/advs.202409173

**Published:** 2025-01-21

**Authors:** Canghai Guan, Jianjun Gao, Xinlei Zou, Wujiang Shi, Yunhe Hao, Yifei Ge, Zhaoqiang Xu, Chengru Yang, Shaowu Bi, Xingming Jiang, Pengcheng Kang, Xiaoxue Xu, Xiangyu Zhong

**Affiliations:** ^1^ General Surgery Department The 2nd Affiliated Hospital of Harbin Medical University 148 Baojian Street Harbin Heilongjiang Province 150086 China; ^2^ The Key Laboratory of Myocardial Ischemia Harbin Medical University Ministry of Education 148 Baojian Street Harbin Heilongjiang 150086 China; ^3^ School of Health Administration Harbin Medical University 148 Baojian Street Harbin Heilongjiang Province 150086 China

**Keywords:** circCPSK6, circular RNA, intrahepatic cholangiocarcinoma, NF‐κB, ubiquitination

## Abstract

Intrahepatic cholangiocarcinoma (ICC), a formidable challenge in oncology, demands innovative biomarkers and therapeutic targets. This research highlights the importance of the circular RNA (circRNA) circPCSK6 and its peptide derivative circPCSK6‐167aa in ICC. CircPCSK6 is significantly downregulated in both ICC patients and mouse primary ICC models, and its lower expression is linked to adverse prognosis, highlighting its pivotal role in ICC pathogenesis. Functionally, this study elucidates the regulatory effect of circPCSK6‐167aa on IκBα ubiquitination within the NF‐κB pathway, which is mediated by its competitive binding to the E3 ligase RBBP6. This complex interaction leads to reduced activation of the NF‐κB pathway, thereby curbing tumor cell proliferation, migration, invasion, stemness, and hepatic‐lung metastasis in vivo. This groundbreaking discovery expands the understanding of circRNA‐driven tumorigenesis through atypical signaling pathways. Additionally, this investigation identified EIF4A3 as a detrimental regulator of circPCSK6, exacerbating ICC malignancy. Importantly, by leveraging patient‐derived xenograft (PDX), organoids, and organoid‐derived PDX models, higher levels of circPCSK6‐167aa enhance sensitivity to gemcitabine, indicating its potential to improve the effectiveness of chemotherapy. These insights emphasize the therapeutic promise of targeting circPCSK6‐167aa, offering vital biological insights and clinical directions for developing cutting‐edge therapeutic approaches, thus revealing innovative strategies and targets for future treatments.

## Graphical abstract

This study reveals the crucial roles of the circular RNA circPCSK6 and its derivative peptide circPCSK6‐167aa in combating intrahepatic cholangiocarcinoma. These findings highlight how these biomolecules modulate the NF‐κB pathway to inhibit tumor progression and enhance chemotherapy efficacy, suggesting new therapeutic targets.

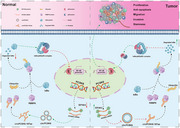

## 1 Introduction

Intrahepatic cholangiocarcinoma (ICC) presents formidable challenges in modern medical treatment and forecasting outcomes.^[^ ^1^ ^]^ The prevalence of ICC is increasing, compounded by the absence of robust early diagnostic techniques,^[^ ^2^ ^]^ and many patients are diagnosed at advanced stages.^[^ ^3^ ^]^ Conventional therapeutic strategies such as surgery, radiotherapy, and chemotherapy,^[^ ^4^ ^]^ while extending the survival period to some extent, still result in poor overall prognosis.^[^ ^2^, ^5^ ^]^ Therefore, identifying new biomarkers and therapeutic targets is crucial for enhancing the clinical outcomes of ICC patients.^[^ ^6^ ^]^ Recently, circular RNA (circRNA), a unique type of non‐coding RNA known for its potential to regulate gene expression across various cancers, has garnered significant interest.^[^ ^7^ ^]^ Owing to their unique circular structure, circRNAs are more stable than their linear counterparts are, making them promising candidates for both biomarkers and therapeutic interventions.^[^ ^8^ ^]^ CircRNAs participate in cancer progression through various mechanisms, including acting as sponges for microRNAs,^[^ ^9^ ^]^ affecting the expression of parent genes,^[^ ^10^ ^]^ and encoding peptides or proteins involved in regulating cellular functions.^[^ ^11^ ^]^ Although some studies have revealed the significant role of circRNAs in various cancers,^[^ ^12^ ^]^ their specific functions and mechanisms in ICC are not well understood, prompting our investigation into a particular circRNA, circPCSK6 (hsa_circ_0037100), through comprehensive bioinformatics analysis and experimental validation.

Our bioinformatics approach and quantitative analyses of patient tissue samples revealed a significant downregulation of circPCSK6 in tumor tissues compared with that in their normal counterparts, which was correlated with poorer prognoses in ICC patients. We confirmed the circular structure and localization characteristics of circPCSK6 through techniques such as Sanger sequencing, subcellular localization assays, and fluorescence in hybridization (FISH) and subsequently elucidated its involvement in ICC cellular proliferation, anti‐apoptotic effects, migration, invasion, and tumor stemness through cell and animal experiments. Further experiments revealed that circPCSK6 functions through its encoded protein circPCSK6‐167aa, mainly by regulating the NF‐κB signaling pathway. The NF‐κB pathway is a key regulatory pathway in many cancers and is often associated with tumor stemness,^[^ ^13^ ^]^ and anti‐apoptotic processes.^[^ ^14^ ^]^ Our research revealed that circPCSK6‐167aa stabilizes the IκBα protein, preventing its ubiquitination and subsequent degradation, which in turn inhibits NF‐κB activation. These results provide new insights into how circRNAs and their encoded proteins directly participate in intracellular signaling mechanisms to affect tumor biology, highlighting the potential of these molecules as novel cancer treatment targets. Additionally, we explored the regulation of circPCSK6 expression by EIF4A3, an RNA‐binding protein.^[^ ^15^ ^]^ RIP experiments revealed that EIF4A3 significantly enriches circPCSK6. Notably, EIF4A3 expression in ICC cells and tissues is inversely correlated with circPCSK6 levels, suggesting that EIF4A3 might indirectly influence circPCSK6 splicing or stability and thereby its function.

Furthermore, in our pursuit of translational applications, we employed patient‐derived xenograft (PDX) models and organoid cultures to investigate the correlation between circPCSK6‐167aa levels and the responsiveness of ICCs to the chemotherapeutic agent gemcitabine. Our findings indicate that circPCSK6‐167aa could increase the efficacy of chemotherapy, providing a foundation for the clinical application of circRNAs and their encoded proteins and suggesting a strategy to improve therapeutic outcomes through circRNA regulation. In conclusion, this study illuminates the role of circPCSK6 in ICC and highlights its potential as a novel therapeutic target.

## 2 Results

### 2.1 Characterization of circPCSK6 in Intrahepatic Cholangiocarcinoma and its Correlation with Patient PROGNOSIS

**Figure 1:**
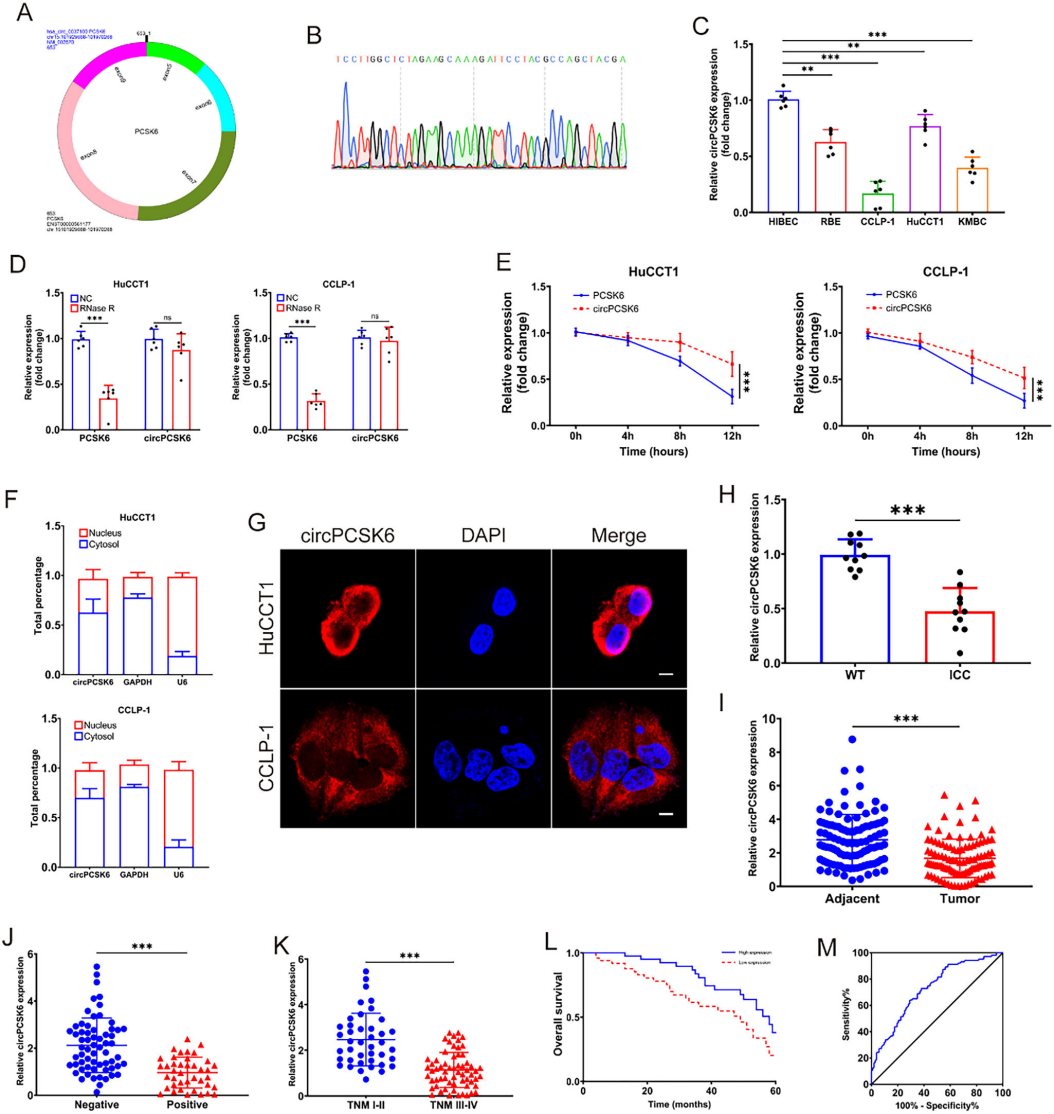
Expression and identification of circPCSK6 in ICC and its correlation with clinicopathological characteristics. A) Schematic representation of the structure of circPCSK6 (hsa_circ_0037100). B) Validation of the back‐splicing junction site of circPCSK6 in ICC cells through Sanger sequencing. C) qRT‐PCR detection of circPCSK6 expression in ICC cells (*n* = 6). D) Evaluation of the stability of circPCSK6 and its linear counterpart PCSK6 mRNA in HuCCT1 and CCLP‐1 cells through RNase R resistance assays (*n* = 6). E) The relative abundance of circPCSK6 and PCSK6 mRNAs after actinomycin D (10 µg mL^−1^) treatment was detected via qRT‐PCR (*n* = 6). F) The subcellular distribution of circPCSK6 was determined by nuclear/cytoplasmic separation via qRT‐PCR (*n* = 6). G) Localization of circPCSK6 in HuCCT1 and CCLP‐1 cells detected by FISH. Scale bar: 5 µm. H) The relative expression level of circPCSK6 in wild‐type (WT) and primary ICC mouse tissues was determined via qRT‐PCR (*n* = 10). I) The relative expression levels of circPCSK6 in ICC tissues compared with those in adjacent normal tissues were determined via qRT‐PCR (*n* = 103). J) The correlation between circPCSK6 expression and different tumor TNM stages was analyzed (*n* = 103). K) Relationship between circPCSK6 expression and lymph node metastasis (*n* = 103). L) OS of patients with high and low circPCSK6 expression. M) ROC curve analysis was conducted to determine the prognostic potential of circPCSK6 expression levels in ICC. Data in (C–F) were presented by two‐way ANOVA test. Data in (H‐K) were presented by two‐tailed Student's *t*‐test. Data in (L) was presented by Kaplan–Meier. **p < 0.01; ***p < 0.001. Data are represented as mean ± SD.

Researchers have employed advanced bioinformatics tools to conduct a thorough analysis of the GSE181523 database, highlighting the pivotal role of circular RNAs in intrahepatic cholangiocarcinoma. The expression profile of hsa_circ_0037100 was significantly lower in ICC tissue samples than in normal tissue samples, as visibly demonstrated by volcano plots and heatmaps (Figure S1A,B, Supporting Information). Advanced computational prediction algorithms delineated the origin of hsa_circ_0037100, pinpointing its genesis through back‐splicing of exons five to night within the PCSK6 gene locus (**Figure** **1**A; Figure S1C, Supporting Information). This origin was corroborated within the ICC cellular environment through the use of divergent primers and Sanger sequencing, confirming the specific back‐splicing site (Figure 1B). Across four distinct ICC cell lines, circPCSK6 exhibited uniformly subdued expression compared with the baseline expression observed in normal biliary epithelial cells (HIBECs) (Figure 1C). The HuCCT1 and CCLP‐1 cell lines, which presented the highest and lowest circPCSK6 expression, respectively, were selected for RNase R treatment, and circPCSK6 was more resistant to degradation than its parental mRNA, PCSK6 (Figure 1D). Furthermore, under Act D treatment, the degradation kinetics of circPCSK6 were significantly slower than those of PCSK6 mRNA, underscoring its enhanced stability (Figure 1E). Subcellular localization investigations, including fluorescence in situ hybridization, revealed that circPCSK6 was predominantly located in the cytoplasm, with relatively minor nuclear accumulation (Figure 1F,G). qRT‐PCR analysis, which was conducted after inducing ICC via the AKT/Notch intracellular domain (NICD) in primary mouse models, revealed significant downregulation of circPCSK6 in ICC compared with that in wild‐type (WT) liver tissue (Figure 1H; Figure S1D, Supporting Information). Further quantitative analyses of patient‐derived ICC tumor tissues in conjunction with their matched normal counterparts revealed a profound downregulation of circPCSK6 expression within the tumor (Figure 1I). Subsequent stratification on the basis of circPCSK6 expression levels revealed markedly lower levels in tumor tissues from patients with lymph node metastasis and advanced TNM stages (Figure 1J,K). The prognostic significance of reduced circPCSK6 expression was underscored by receiver operating characteristic (ROC) and Kaplan–Meier analyses, which revealed that it was correlated with unfavorable patient outcomes (Figure 1L,M). Multivariate Cox regression analysis further identified TNM stage as an independent prognostic determinant for patients with decreased circPCSK6 expression (**Tables** **1** and **2**). Consequently, the attenuated expression of circPCSK6 is recognized as a significant indicator of ICC pathogenesis, indicating a dire progression marked by aggressive disease development and compromised clinical prognosis.

**Table 1:** Correlations between circPCSK6 expression and clinicopathological characteristics of ICC patients.

Clinicopathological parameters	Total Cases(*n* = 103)	circPCSK6 expression		*p* value
		Low (*n* = 51)	High (*n* = 52)	
Age (years)				0.9424
<60	36	18	18	
≥60	67	33	34	
Gender				0.9176
Male	49	24	25	
Female	54	27	27	
Smoking				0.6106
No	58	30	28	
Yes	45	21	24	
Alcoholic				0.9073
No	60	30	30	
Yes	43	21	22	
TNM stage				**0.0008**
I‐II	41	12	29	
III‐IV	62	39	23	
Lymph node invasion				**0.0002**
Negative	63	22	41	
Positive	40	29	11	
HBV infection				0.1221
Negative	70	31	39	
Positive	33	20	13	
Serum CEA (ng mL^−1^)				0.9374
>5	63	31	32	
≤5	40	20	20	
Serum CA19‐9 (U mL^−1^)				0.9143
>37	56	28	28	
≤37	47	23	24	

Bold values indicate *p* < 0.05.

**Table 2:** Univariate and multivariate analyses of the overall survival of ICC patients.

Variables	Univariate analysis			Multivariate analysis		
	HR	95% CI	*p* value	HR	95% CI	*p* value
Age (years)						
≥60 vs <60	0.982	0.532–1.812	0.952			
Gender						
Male vs Female	0.635	0.348–1.160	0.140			
Smoking						
Yes vs No	1.237	0.680–2.253	0.486			
Alcoholic						
Yes vs No	0.932	0.508–1.712	0.921			
HBV infection						
Positive vs Negative	1.321	0.710–2.458	0.380			
Serum CEA (ng mL^−1^)						
>5 vs ≤5	0.925	0.498–1.716	0.804			
Serum CA19‐9 level (U mL^−1^)						
>37 vs ≤37	0.833	0.459–1.510	0.547			
Lymph node invasion						
Positive vs Negative	2.052	1.115–3.774	0.021*	0.766	0.333–1.761	0.053
TNM stage						
III‐IV vs I‐II	1.920	1.025–3.596	0.042*	1.563	0.751–3.255	**0.023**
CircPCSK6 expression						
Low vs High	0.488	0.265–0.898	0.021*	1.467	0.702–3.069	**0.031**

Bold values indicate *p* < 0.05.

### 2.2 Regulatory Effects of circpcsk6 on the Malignant Biological Behaviors of ICC Cells

**Figure 2:**
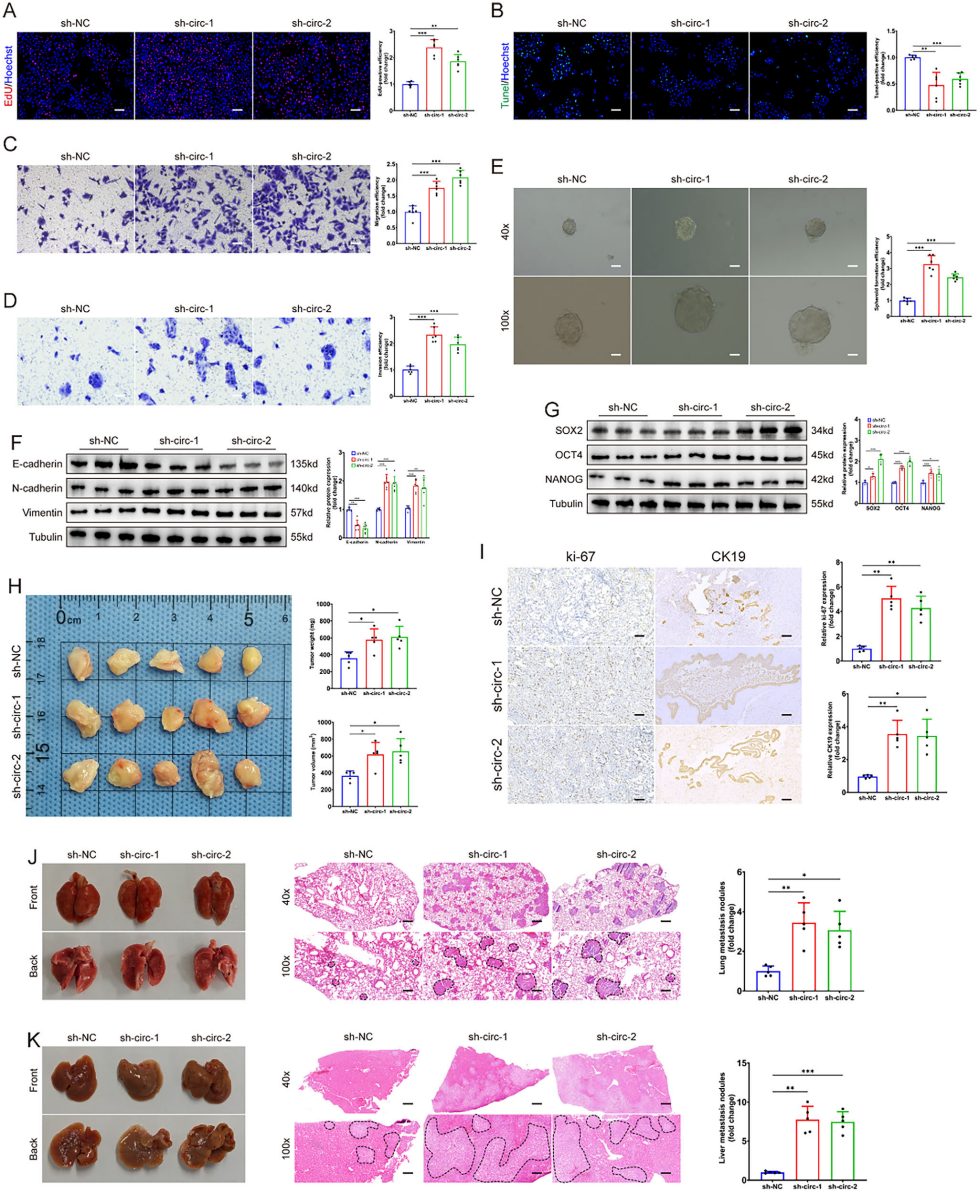
Knockdown of circPCSK6 inhibits ICC malignant behaviors in vitro and in vivo. A) Changes in HuCCT1 cell proliferation were detected via EdU assays (*n* = 6). Scale bar: 100 µm. B) Changes in the apoptosis of HuCCT1 cells were assessed via Tunel assays (*n* = 6). Scale bar: 100 µm. C,D) The migration and invasion potentials of HuCCT1 cells were measured via Transwell migration and invasion assays, respectively (*n* = 6). Scale bar: 50 µm. E) Tumor sphere formation assays were used to evaluate the sphere formation efficiency of HuCCT1 cells (*n* = 6). Scale bar: 100 µm. F,G) Western blot analysis focused on changes in the expression of EMT‐related and tumor stem cell‐related markers (*n* = 6). H) Weights and volumes of subcutaneous tumors after circPCSK6 was silenced (*n* = 5). I) Ki‐67 and CK19 staining of subcutaneous tumor tissues from each group (*n* = 5). Scale bar: 50 µm. J) Images and corresponding H&E staining of lung metastases after circPCSK6 was silenced (*n* = 6). Scale bar: 100 µm. K) Representative images of liver metastases after circPCSK6 knockdown (*n* = 6). Scale bar: 100 µm. Data in (A–E,H–K) were presented by one‐way ANOVA test. Data in (F,G) were presented by two‐way ANOVA test. *p < 0.05; **p < 0.01; ***p < 0.001. Data are represented as mean ± SD.

Upon confirming the increased relative abundance of circPCSK6 in HuCCT1 cells, its expression was deliberately reduced via shRNA lentiviral vectors, specifically sh‐circ‐1 and sh‐circ‐2, which were selected for their effectiveness (Figure S1E, Supporting Information). EdU assays revealed a significant increase in DNA replication and cellular proliferation following the knockdown of circPCSK6 (**Figure** **2**A). Tunel assays revealed a concomitant decrease in cellular apoptosis associated with reduced circPCSK6 expression (Figure 2B). Transwell assays further demonstrated the increased migratory and invasive capabilities of HuCCT1 cells with circPCSK6 suppression (Figure 2C,D). Subsequent sphere formation assays revealed increased acquisition of stem cell‐like attributes within ICC cells upon circPCSK6 silencing (Figure 2E). Western blot analyses revealed changes in the expression of epithelial‐mesenchymal transition (EMT)‐associated molecules (E‐cadherin, N‐cadherin, and Vimentin) and stemness markers (SOX2, OCT4, and NANOG) resulting from circPCSK6 downregulation, which play crucial roles in driving tumor invasiveness and regulating stem cell dynamics (Figure 2F,G). In vivo studies using subcutaneous tumorigenesis and metastasis models in nude mice revealed a marked increase in tumor volume and a greater incidence of liver and lung metastases following circPCSK6 knockdown. These findings were supported by increased immunopositivity for ki‐67 and CK19, alongside elevated levels of EMT‐associated proteins and stem cell markers (Figure 2H–K; Figure S2A,B, Supporting Information). Moreover, when primary ICC tissues from mice were categorized on the basis of circPCSK6 expression levels, those with lower circPCSK6 expression presented larger primary tumor foci and a higher positive rate for CK19 (Figure S2C,D, Supporting Information). In summary, these observations collectively suggest a pivotal role of circPCSK6 in dictating the proliferative, anti‐apoptotic, migratory, invasive, and stem‐like traits of ICC cells, alongside its contributory role in the augmentation of subcutaneous tumor growth and metastatic progression in murine models.

### 2.3 CircPCSK6‐167aa, a Novel Protein of 167 Amino Acids Encoded by circPCSK6

**Figure 3:**
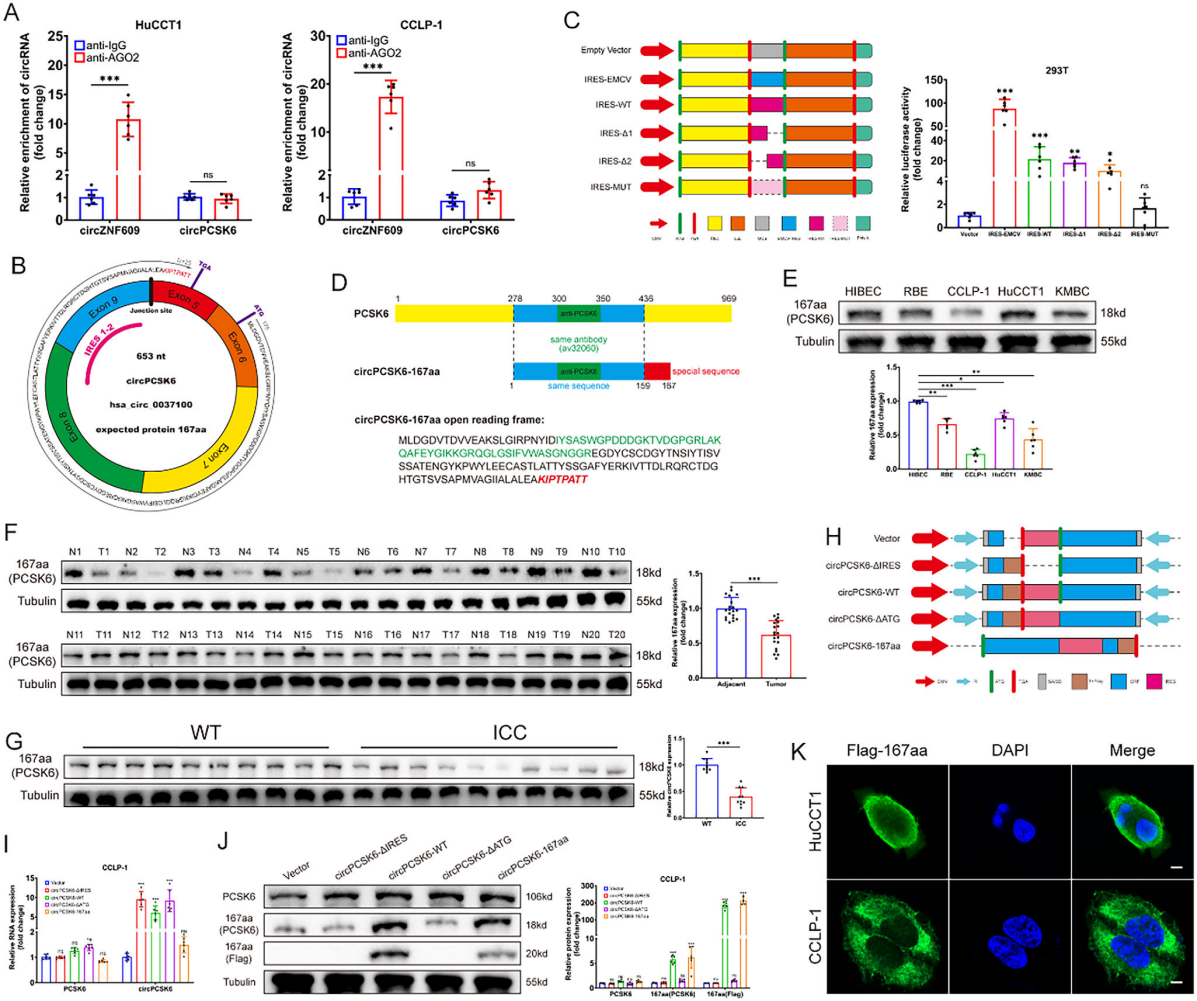
CircPCSK6 encodes a novel 167‐amino acid protein in ICC, named circPCSK6‐167aa. A) RIP assays verified the association between circPCSK6 and AGO2, with circZNF609 used as a positive control (*n* = 6). B) Schematic diagram illustrating the putative ORF in circPCSK6 and the amino acid sequence encoded by the putative ORF. C) IRES sequences of different truncations of circPCSK6 were cloned from Rluc and Luc reporters, and luciferase activity in different vectors was measured via luciferase reporter assays, with EMCV‐IRES as a positive control (*n* = 6). D) An immunogenic sequence map was generated by antibodies recognizing both the PCSK6 and circPCSK6‐167aa proteins, with the green sequence being the recognized sequence and the red sequence being the unique amino acid sequence of circPCSK6‐167aa. E) Western blot detection of circPCSK6‐167aa expression in different ICC cell lines (*n* = 6). F) Protein expression levels of circPCSK6‐167aa in ICC tissues compared with adjacent normal tissues (*n* = 20). G) Comparison of circPCSK6‐167aa protein expression levels in the liver tissues of primary ICC mice and wild‐type mice (*n* = 10). H) Construction of five circPCSK6‐related vectors: empty vector; circPCSK6‐WT: circPCSK6 sequence with a 3×Flag tag cloned into a CMV‐driven expression vector; circPCSK6‐ΔATG: mutated start codon (ATG – ACG) of the circPCSK6 sequence with a 3×Flag tag cloned and inserted into an overexpression vector; circPCSK6‐ΔIRES: IRES binding site‐deleted circPCSK6 sequence with a 3×Flag tag cloned into an overexpression vector; circPCSK6‐167aa: linearized circPCSK6‐167aa sequence with a 3×Flag tag cloned and inserted into a CMV‐driven expression vector. I) Relative expression of circPCSK6 and PCSK6 mRNAs measured by qRT‐PCR after overexpression of the above vectors (*n* = 6). J) Western blot detection of circPCSK6‐167aa expression efficiency after overexpression of the above vectors in ICC (*n* = 6). K) IF detection of the cellular localization of exogenously expressed circPCSK6‐167aa. Scale bar: 5 µm. Data in (A,I,J) were presented by two‐way ANOVA test. Data in (C) was presented by one‐way ANOVA test. Data in (F,G) were presented by two‐tailed Student's *t*‐test. *p < 0.05; **p < 0.01; ***p < 0.001. Data are represented as mean ± SD.

To elucidate the intricate regulatory dynamics orchestrated by circPCSK6 in tumor progression, we first revisited its predominantly cytoplasmic localization. Conventional wisdom posits that cytoplasmic circular RNAs primarily engage in molecular interactions,^[^ ^16^ ^]^ often functioning as competitive endogenous RNAs (ceRNAs) to sequester microRNAs,^[^ ^17^ ^]^ thereby modulating gene expression, which influences tumor progression.^[^ ^18^ ^]^ However, the use of the circInteractome online prediction tool revealed an intriguing finding: circPCSK6 does not engage in binding interactions with the AGO2 protein (Figure S3A, Supporting Information), a pivotal constituent of the RNA‐induced silencing complex (RISC) central to microRNA‐mediated mechanisms.^[^ ^19^ ^]^ RIP assays revealed a stark departure from the binding affinity observed for circZNF609 (**Figure** **3**A). Our previous studies demonstrated that circZNF609 can bind to AGO2,^[^ ^20^, ^21^ ^]^ suggesting that the tumor‐suppressive function of circPCSK6 may diverge from the canonical ceRNA paradigm.

Further analysis revealed an open reading frame (ORF) within circPCSK6 that encodes a novel 167‐amino acid protein, named circPCSK6‐167aa or simply 167aa. The sequence includes two internal ribosome entry sites (IRESs), indicating a translation mechanism independent of the conventional 5′ cap structure, relying instead on these IRES elements (Figure 3B). Functional validation of these IRES motifs (488‐637 and 523–651 nt) was confirmed through luciferase reporter assays, which revealed significant IRES activity (Figure 3C). We identified an antibody that recognizes both the PCSK6 and circPCSK6‐167aa proteins (Figure 3D). Further validation efforts revealed a marked downregulation of circPCSK6‐167aa in ICC cells and tissues, which was consistent with previous qRT‐PCR results indicating a decrease in circPCSK6 levels (Figure 3E–G).

To investigate the pivotal role of circPCSK6‐167aa in mediating the tumor‐suppressive effects of circPCSK6, we engineered five distinct 3×Flag‐tagged vectors: an empty vector (vector), a vector bearing a 3×Flag tag preceding the ORF stop codon (circPCSK6‐WT), a vector harboring a mutated ORF start codon (circPCSK6‐ΔATG), a vector excising the sequence impeding circularization (circPCSK6‐ΔIR), and a vector capable of transcribing a linearized full‐length ORF sequence (circPCSK6‐167aa) (Figure 3H). The experiments demonstrated that the mRNA and protein levels of PCSK6 remained stable; however, circPCSK6‐WT and circPCSK6‐ΔATG effectively increased circPCSK6 RNA levels (Figure 3I). Unlike circPCSK6‐ΔATG, which did not increase the expression of 167 aa, both circPCSK6‐WT and circPCSK6‐167aa significantly increased the expression of 167 aa (Figure 3J). A proteomic analysis conducted on the cytoplasm of CCLP‐1 and 293T cells transfected with circPCSK6‐WT and control vectors, followed by SDS‐PAGE, revealed a distinct protein band near 18 kDa (Figure S3B, Supporting Information). Moreover, the externally expressed circPCSK6‐167aa was predominantly localized within the cytoplasm (Figure 3K). These findings confirm the successful validation of a novel peptide encoded by circPCSK6, marked by its unique amino acid sequence (KIPTPATT) and suggest its potential role in the modulation of tumor biology through mechanisms distinct from typical ceRNA interactions.

### 2.4 Tumor Growth and Metastasis Suppression by circPCSK6‐167aa

**Figure 4:**
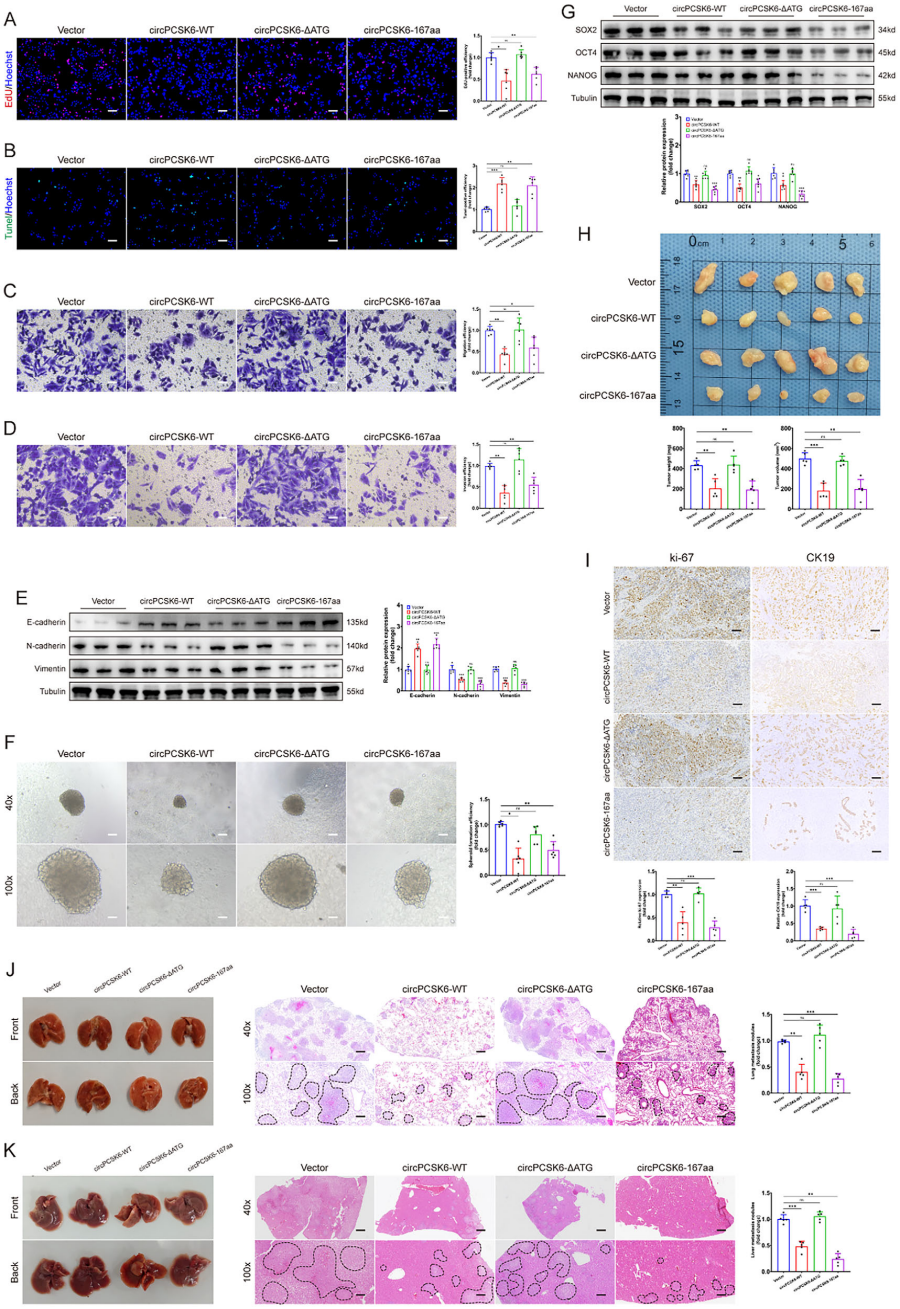
The anti‐carcinogenic effect of circPCSK6 in CCLP‐1 is mediated by its encoded protein circPCSK6‐167aa. A,B) Proliferation and apoptosis abilities of CCLP‐1 detected by EdU and Tunel assays, respectively, after transfection with empty vector, circPCSK6‐WT, circPCSK6‐ΔATG, and circPCSK6‐167aa (*n* = 6). Scale bar: 100 µm. C,D) Migration and invasion efficiencies of cells detected by transwell assays after transfection with the four vectors (*n* = 6). Scale bar: 50 µm. E) Western blot analysis was used to examine the impact of the four vectors on EMT marker expression (*n* = 6). F,G) The tumorigenicity and stemness of CCLP‐1 were validated by tumor sphere formation assays and stemness marker detection (*n* = 6). Scale bar: 100 µm. H) Nude mouse xenografts formed from CCLP‐1 cells transfected with empty vector, circPCSK6‐WT, circPCSK6‐ΔATG, or circPCSK6‐167aa (*n* = 5). I) IHC detection of ki‐67‐ and CK19‐positive rates in subcutaneous tumor tissues after transfection with the four vectors (*n* = 5). Scale bar: 100 µm. J,K) Lung and liver metastasis of CCLP‐1 in vivo after transfection with the four overexpression vectors (*n* = 6). Scale bar: 100 µm. Data in (A–D,F,H–K) were presented by one‐way ANOVA test. Data in (E,G) were presented by two‐way ANOVA test. *p < 0.05; **p < 0.01; ***p < 0.001. Data are represented as mean ± SD.

A series of cellular and in vivo investigations revealed that the enforced expression of circPCSK6 and its encoded 167aa protein resulted in profound inhibition of tumor cell proliferation and was associated with the promotion of apoptotic events. Conversely, the vector with a mutagenized ORF start codon, circPCSK6‐ΔATG, did not have any discernible effect on the proliferative or apoptotic dynamics of CCLP‐1 cells (**Figure** **4**A,B). Further insights from transwell assays and assessments of EMT marker proteins underscored the potent inhibitory effects of circPCSK6‐167aa on the migratory, invasive, and EMT‐mediated processes inherent to ICC cells (Figure 4C–E). Sphere formation assays and western blot analyses targeting stem cell‐related proteins provided corroborative evidence, confirming the profound ability of circPCSK6‐167aa to curtail the stem cell‐like attributes of tumor cells (Figure 4F,G).

Moreover, the establishment of stable cell lines harboring overexpression vectors facilitated a compelling demonstration in a murine xenograft model. Augmented expression of circPCSK6 and circPCSK6‐167aa markedly reduced the volume and mass of subcutaneous tumors, concomitant with a discernible suppression of ki‐67 and CK19 expression (Figure 4H,I). Concurrently, the incidence of liver and lung metastases was significantly reduced in both the circPCSK6‐WT and circPCSK6‐167aa groups, as were the expression of (N‐cadherin and Vimentin) and stem cell markers, compared with their control and non‐coding counterparts (Figure 4J,K; Figure S4A,B, Supporting Information). Collectively, these findings underscore the pivotal role of circPCSK6, which is mediated through its encoded 167aa protein, in orchestrating a multifaceted anticancer regimen targeting the growth and metastatic dissemination of ICC.

### 2.5 Regulation of IκBα Ubiquitination in the NF‐κB Pathway by the E3 Ligase RBBP6 via circPCSK6‐167aa

**Figure 5:**
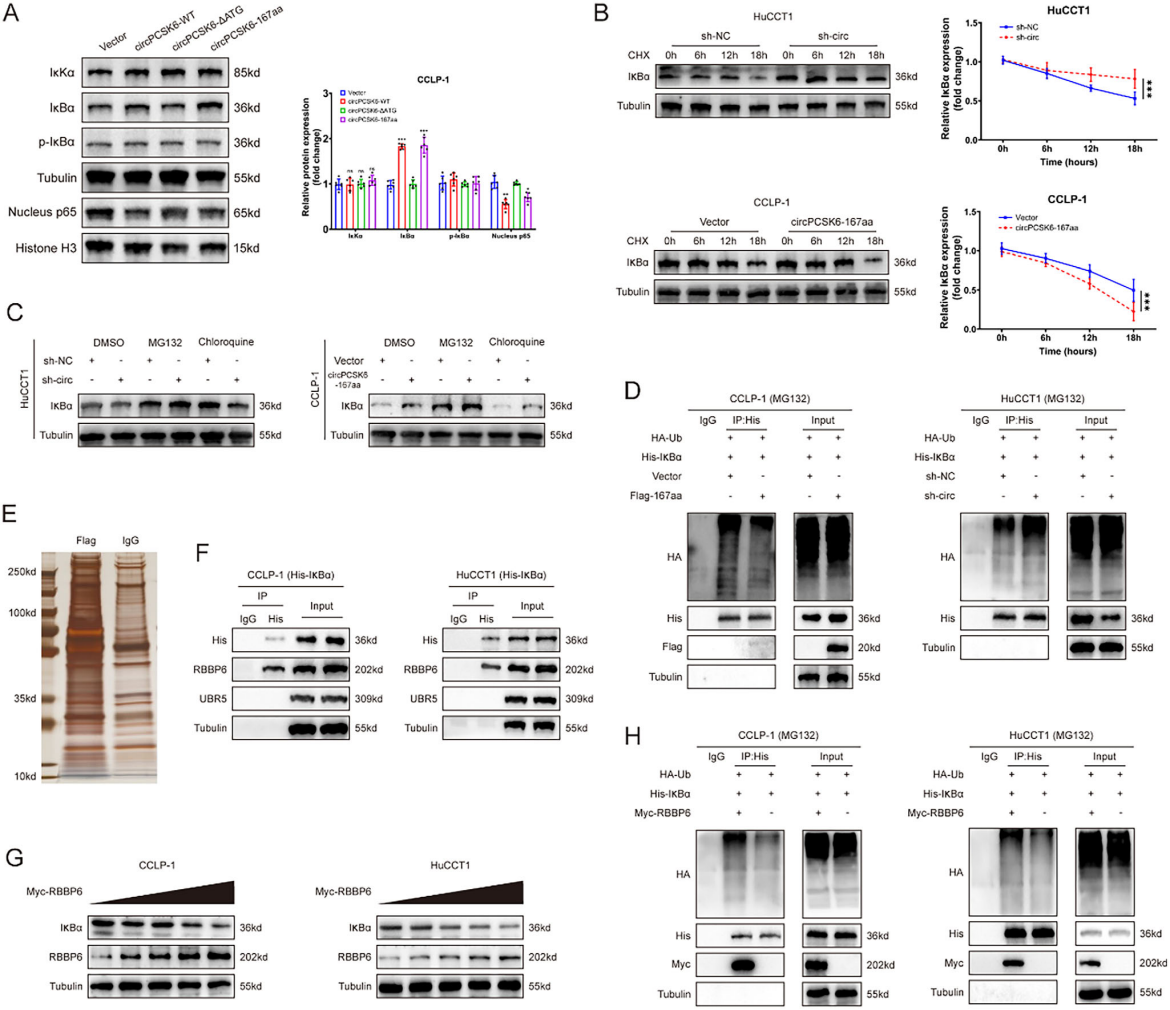
CircPCSK6‐167aa interacts with RBBP6 to promote the ubiquitin‐dependent degradation of IκBα. A) Expression of NF‐κB signaling pathway‐related proteins after overexpression of the four vectors (*n* = 6). B) Evaluation of IκBα protein expression in HuCCT1 and CCLP‐1 cells after circPCSK6‐167aa was silenced or overexpressed with CHX treatment over time (*n* = 6). C) Changes in IκBα protein levels in different groups after treatment with the proteasome inhibitor MG132 and the lysosome inhibitor chloroquine. D) Co‐IP experiments were used to investigate the ubiquitination of IκBα after circPCSK6‐167aa regulation. E) Co‐IP experiments in cells overexpressing circPCSK6‐167aa via anti‐Flag and anti‐IgG, with silver staining of the co‐precipitated products. F) Co‐IP validation of the interaction between IκBα and UBR5 or RBBP6. G) Western blot analysis of the relative expression of RBBP6 and IκBα after gradient overexpression of RBBP6. H) Co‐IP detection of the regulatory effect of RBBP6 overexpression on the level of ubiquitinated IκBα. Data in (A, B) were presented by two‐way ANOVA test. *p < 0.05; **p < 0.01; ***p < 0.001. Data are represented as mean ± SD.

To investigate the tumor‐suppressive roles governed by circPCSK6‐167aa in ICC, we utilized RNA‐seq and bioinformatics analyses to explore cells with elevated circPCSK6‐167aa levels (Figure S5A,B, Supporting Information). These studies revealed a significant enrichment of the NF‐κB signaling pathway (Figure S5C,D, Supporting Information). Despite the absence of discernible alterations in IκKα and p‐IκBα expression, the increased levels of circPCSK6‐WT and circPCSK6‐167aa led to a notable increase in IκBα expression along with a marked reduction in p65 in the nucleus (**Figure** **5**A). A reduction in circPCSK6 produced opposite effects (Figure S5E, Supporting Information). Intriguingly, these findings suggest a potential regulatory role of circPCSK6‐167aa in dampening the NF‐κB pathway, likely through a mechanism unrelated to IκBα phosphorylation. Validation by qRT‐PCR confirmed that circPCSK6‐167aa modulates IκBα expression post‐transcriptionally, without affecting IκBα mRNA levels (Figure S5F, Supporting Information).

Further delineation of the regulatory effects was conducted through CHX‐mediated inhibition of new protein synthesis, which revealed that the overexpression of circPCSK6‐167aa slowed IκBα protein degradation, whereas the silencing of circPCSK6 accelerated this process (Figure 5B). Mechanistic studies using the proteasome inhibitor MG132 and lysosome inhibitor Chloroquine identified the proteasomal degradation pathway as the main pathway affected by circPCSK6‐167aa in the regulation of IκBα protein stability (Figure 5C).

Co‐IP analysis revealed that both increasing and decreasing circPCSK6‐167aa levels affected the ubiquitination of IκBα (Figure 5D). Subsequent IP‐MS analyses, marked by flag‐tagging, revealed 219 potential interactors with circPCSK6‐167aa, highlighting the E3 ligases RBBP6 and UBR5 as key candidates for further examination (Figure 5E; Figure S5G, Supporting Information). Co‐IP experiments confirmed the binding affinity between IκBα and RBBP6, with minimal interaction observed with UBR5 (Figure 5F). Additional evidence from the GEPIA database revealed an unusual increase in RBBP6 levels in cholangiocarcinoma tissues, along with reduced IκBα levels (Figure S5H, Supporting Information). Through a gradual increase in RBBP6 expression, we observed a corresponding gradual decrease in IκBα expression (Figure 5G). Furthermore, the results of Co‐IP confirmed that RBBP6 facilitated the ubiquitination of IκBα (Figure 5H). These experiments confirmed that RBBP6 is the authentic E3 ligase for IκBα and orchestrates the ubiquitination of IκBα.

### 2.6 Inhibition of Malignant Biological Behavior in ICC Through Competitive Binding of circPCSK6‐167aa to RBBP6, Leads to Reduced IκBα Ubiquitination

**Figure 6:**
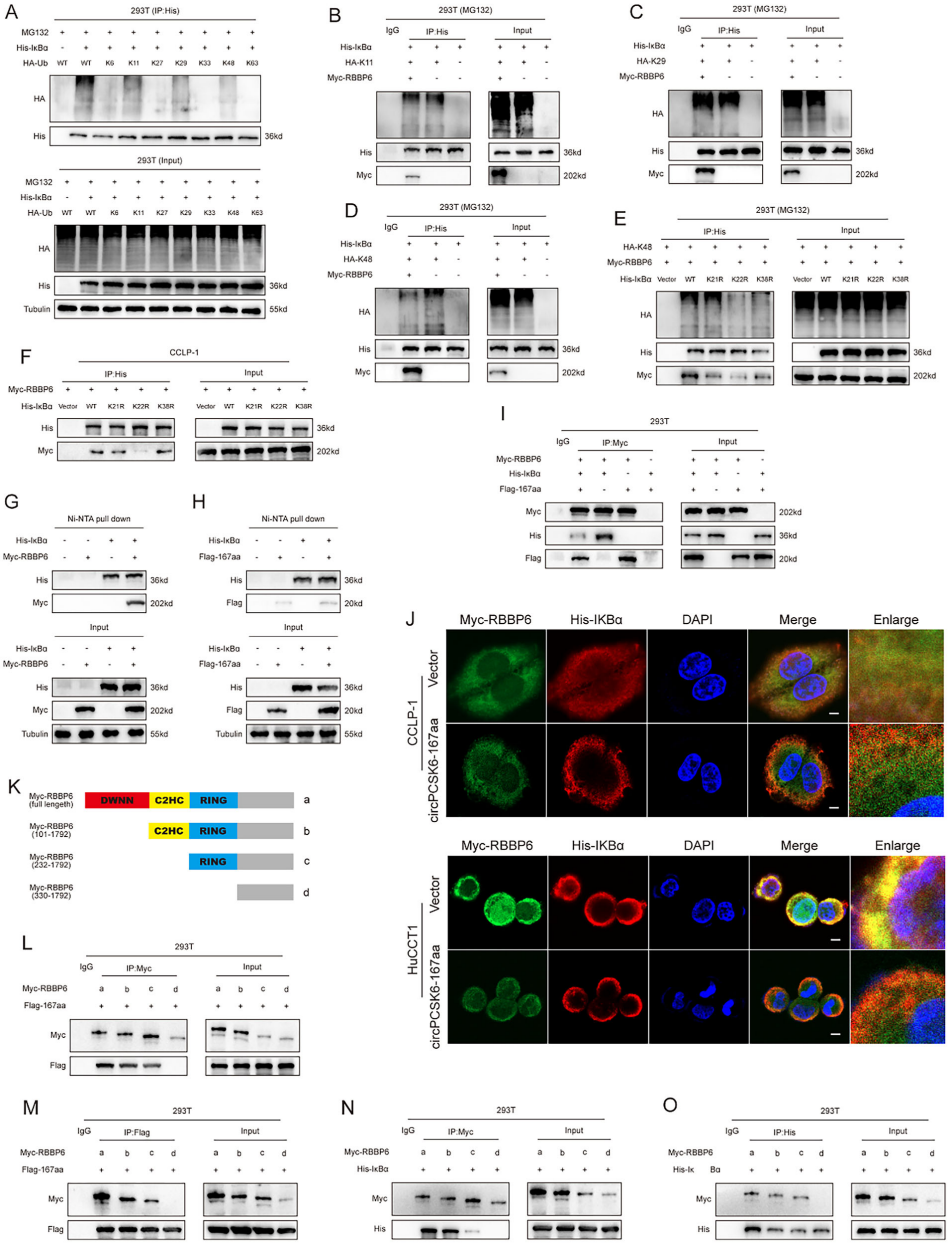
CircPCSK6‐167aa competes with RBBP6 to inhibit the K48‐linked ubiquitination of IκBα. A) Overexpression of specific Ub wild‐type and single lysine‐retaining mutant plasmids and transfection with His‐IκBα in 293T cells facilitated the detection of K6/K11/K27/K29/K33/K48/K63‐linked ubiquitination. B‐D) K11‐linked, K29‐linked, and K48‐linked ubiquitination of IκBα was detected after the upregulation of RBBP6. E) IκBα ubiquitination levels were detected after co‐transfection of three IκBα lysine single mutant vectors with Ub‐K48 in only 293T cells. F) Co‐IP was used to detect the interaction between three IκBα lysine single mutant vectors and RBBP6. G) Ni‐NTA pull‐down and western blot were performed to evaluate the interaction between IκBα and RBBP6 in 293T cells transfected with His‐IκBα and Myc‐RBBP6. H) Ni‐NTA pull‐down confirmed the interaction between His‐IκBα and circPCSK6‐167aa. I) Co‐IP was used to detect the interaction between Myc‐RBBP6 and His‐IκBα in 293T cells transfected with specific vectors. J) IF confirmed the effect of circPCSK6‐167aa on the interaction between endogenous RBBP6 and IκBα in CCLP‐1 and HuCCT1. Scale bar: 5 µm. K) Schematic diagram of RBBP6 proteins with different domains and the Myc‐tagged truncation vectors used in the study. L,M) Co‐IP experiments detecting the specific binding sites of RBBP6 with circPCSK6‐167aa. *n*, O) Co‐IP detection of the interaction between His‐IκBα and specific RBBP6 vectors after co‐transfection.

In our efforts to delineate the varied landscape of IκBα ubiquitination, we undertook a detailed exploration, beginning with the co‐transfection of vectors carrying single lysine mutations in Ub with IκBα. This revealed a predominance of K11, K29, and K48 ubiquitin chain linkages (**Figure** **6**A). Intriguingly, increased RBBP6 expression selectively potentiated Ub‐K48 type ubiquitination, leaving Ub‐K11 and Ub‐K29 unaffected (Figure 6B–D). To further analyze the complex network of ubiquitination sites on IκBα, we employed MusiteDeep and UbPred to predict and create vectors for targeting putative sites (K21, K22, and K38) (Figure S5I, Supporting Information), which were co‐transfecting these with Ub‐K48 and RBBP6. Our findings indicated that RBBP6 was unable to promote K48 ubiquitination of the IκBα‐K22R mutant, a finding supported by Co‐IP experiments that revealed a reduced interaction between the IκBα‐K22R mutant and RBBP6 (Figure 6E,F).

Furthermore, Ni‐NTA pull‐down assays revealed a significant affinity between IκBα and RBBP6, in contrast with the minimal binding observed with circPCSK6‐167aa (Figure 6G,H). Co‐IP analyses revealed a significant decrease in the interaction between RBBP6 and IκBα following circPCSK6‐167aa upregulation (Figure 6I), a finding further corroborated by immunofluorescence microscopy, which revealed a noticeable reduction in the co‐localization of RBBP6 with IκBα after circPCSK6‐167aa increased (Figure 6J). To explore the dynamics of protein‐protein interactions, we constructed truncation vectors targeting distinct structural domains of RBBP6 (DWNN, C2HC, and RING) (Figure 6K), and Co‐IP identified the RING domain as the principal nexus for the competitive binding of circPCSK6‐167aa and IκBα (Figure 6L–O).

Moreover, rescue experiments provided compelling evidence, revealing the reversal of the tumorigenic effects engendered by increased RBBP6 expression upon circPCSK6‐167aa overexpression (Figure S6A–E, Supporting Information). Conversely, the inhibitory effect of down‐regulating RBBP6 expression was reversed after transfection with sh‐circPCSK6 (Figure S6F–J, Supporting Information). Concurrently, the tumor‐suppressive effects attributed to increased IκBα expression could be nullified by circPCSK6 knockdown but were ineffectual against the IκBα‐K22R variant (Figure S7A–E, Supporting Information). Similarly, while the inhibitory effects of increased IκBα expression on ICC proliferation, anti‐apoptosis, migration, invasion, and tumor stemness could be alleviated by RBBP6 overexpression, the malignant biological behavior precipitated by the IκBα‐K22R variant remained refractory to such intervention (Figure S8A–E, Supporting Information). To further assess whether the overexpression of circPCSK6 and circPCSK6‐167aa can mitigate the oncogenic effect of the activated NF‐κB signaling pathway on ICC, we used TNF‐α as an activator and discovered that the overexpression of circPCSK6 and circPCSK6‐167aa effectively reduced the TNF‐α‐induced malignant biological behavior of tumors (Figure S9A–E, Supporting Information). In summary, circPCSK6‐167aa effectively inhibits the ubiquitination of IκBα by competitively binding to RBBP6 and then orchestrates the inhibition of NF‐κB pathway activation, ultimately preventing the malignant biological behavior of ICC.

### 2.7 EIF4A3 as an Upstream Regulator of circPCSK6 and its Role in Suppressing circPCSK6 Expression

**Figure 7:**
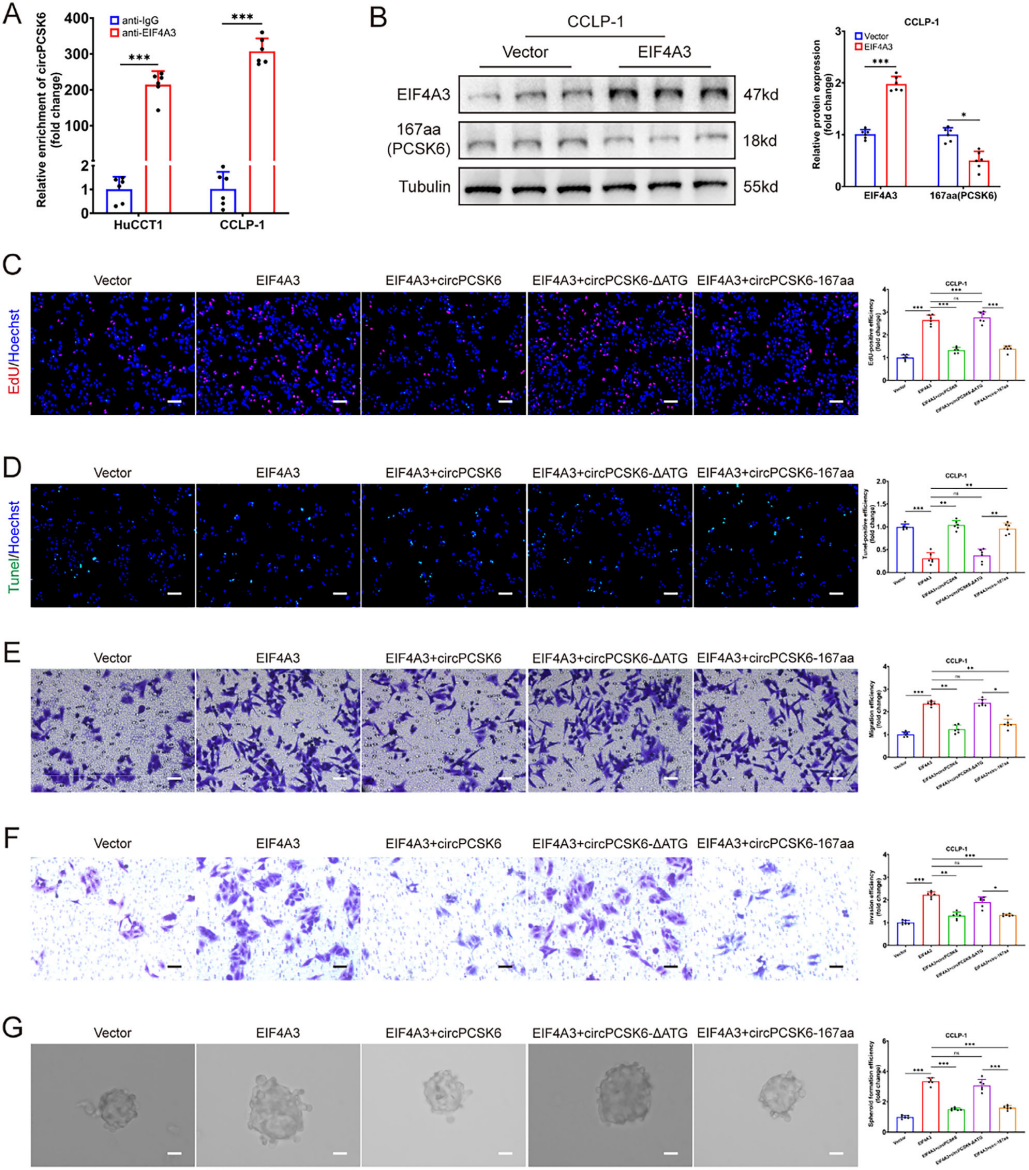
EIF4A3 regulates the production of circPCSK6 and exerts tumor regulatory functions in ICC. A) RIP detection revealed the enrichment of circPCSK6 and EIF4A3 in ICC (*n* = 6). B) Western blot detection was used to quantify the relative expression of EIF4A3 and circPCSK6‐167aa after overexpression of EIF4A3 in CCLP‐1 cells (*n* = 6). C,D) The proliferation and apoptosis abilities of CCLP‐1 cells were assessed via EdU and Tunel assays, respectively, after transfection with the empty vector, EIF4A3, EIF4A3+circPCSK6, EIF4A3+circPCSK6‐ΔATG, and EIF4A3+circPCSK6‐167aa (*n* = 6). Scale bar: 100 µm. E,F) Transwell assays demonstrated that both circPCSK6 and circPCSK6‐167aa can reverse the promotion of the migration and invasion of CCLP‐1 cells induced by the upregulation of EIF4A3, whereas circPCSK6‐ΔATG cannot (*n* = 6). Scale bar: 50 µm. G) Sphere formation assays were used to detect the ability of the three circPCSK6 overexpression vectors to rescue the tumor stemness promoted by EIF4A3 (*n* = 6) Scale bar: 50 µm. G) Sphere formation assays used to detect the ability of the three circPCSK6 overexpression vectors to rescue the tumor stemness promoted by EIF4A3 (*n* = 6). Scale bar: 100 µm. Data in (A,B) were presented by two‐way ANOVA test. Data in (C–G) were presented by one‐way ANOVA test. *p < 0.05; **p < 0.01; ***p < 0.001. Data are represented as mean ± SD.

In our endeavor to decode the complexities of circPCSK6 expression variations within ICCs, our initial analysis focused on the potential impact of transcriptional dynamics within its parental gene, PCSK6, on circPCSK6 expression. Despite detailed experiments involving PCSK6 overexpression and silencing, no significant changes in circPCSK6 expression were observed (Figure S10A, Supporting Information). Consequently, our research emphasis shifted to identifying the molecular mechanisms controlling circRNA splicing.^[^ ^22^ ^]^ Analysis via the circInteractome platform identified EIF4A3 as a potential binding partner that is frequently associated with the flanking regions of circPCSK6 (Figure S3A, Supporting Information). Subsequent RIP assays demonstrated significant enrichment of circPCSK6 within the anti‐EIF4A3 cohort, indicative of a direct interaction between the two entities (**Figure** **7**A).

Further analysis of the GEPIA database in conjunction with western blot analyses unveiled revealed EIF4A3 expression levels in cholangiocarcinoma tissues and ICC cells (Figure S10B–D, Supporting Information). Additional studies established a regulatory role for EIF4A3 in ICC, as shown by the upregulation of circPCSK6‐167aa following EIF4A3 silencing, whereas EIF4A3 overexpression produced the opposite effect, indicating a potential inhibitory role of EIF4A3 in circPCSK6 formation (Figure 7B; Figure S10E, Supporting Information). Functional assays underscored the oncogenic potential of EIF4A3, characterized by its ability to promote ICC cell proliferation, migration, invasion, and tumor stemness, coupled with concomitant suppression of apoptosis. Intriguingly, the adverse effects of EIF4A3 overexpression were mitigated by concurrent overexpression of circPCSK6 or circPCSK6‐167aa, although overexpression of circPCSK6‐ΔATG failed to attenuate the oncogenic activity of EIF4A3 (Figure 7C–G).

### 2.8 Revealing the Role of circPCSK6 in Gemcitabine Resistance in ICC Through Human‐Derived Xenografts and Organoids

**Figure 8:**
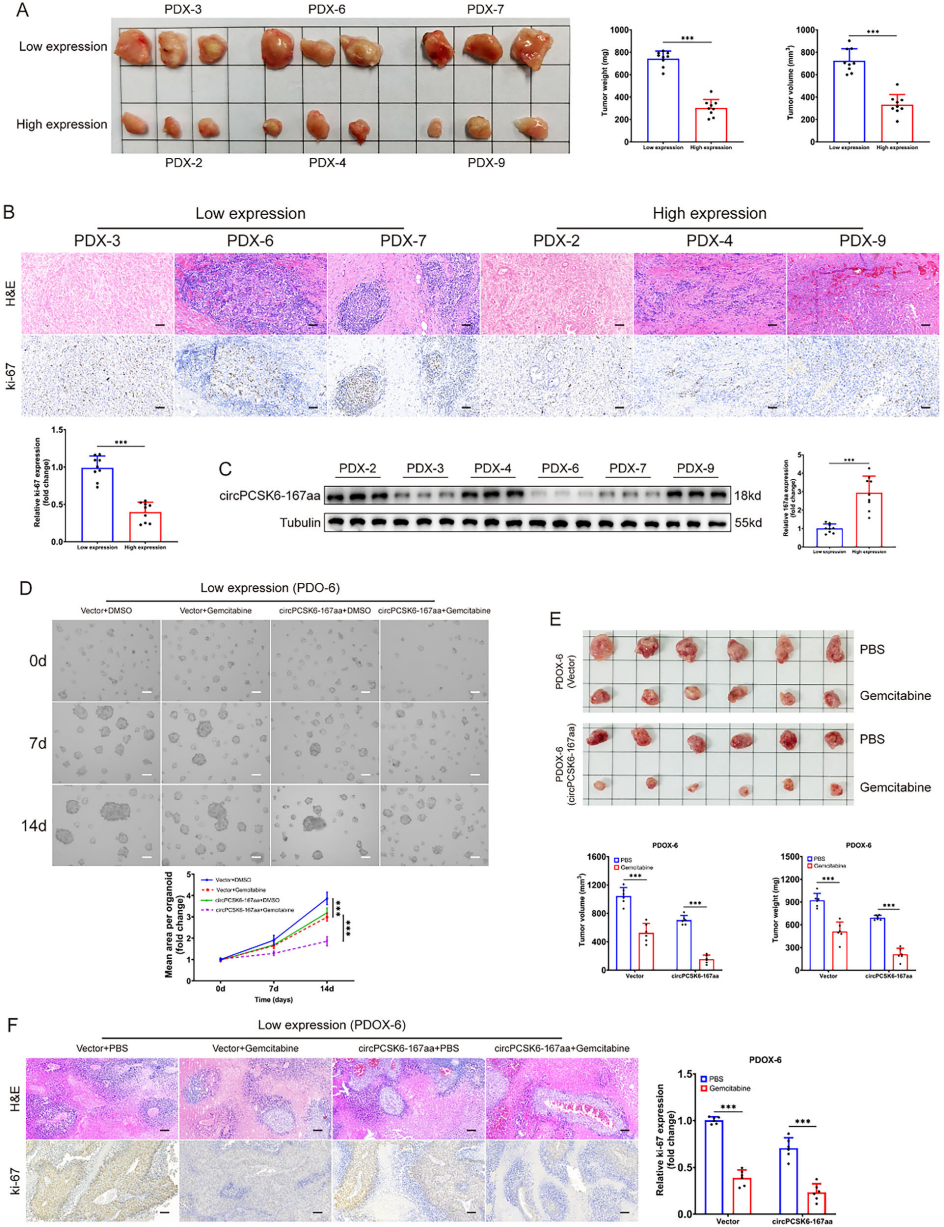
CircPCSK6‐167aa is a potential therapeutic target for ICC patients. A) Patient samples were divided into high‐ and low‐circPCSK6 expression groups on the basis of the median expression level of circPCSK6. PDX models were constructed, and subcutaneous tumors were collected for weight and volume measurements (*n* = 9). B) H&E staining and ki‐67 immunohistochemistry of tumor tissues from different PDX models (*n* = 9). Scale bar: 100 µm. C) Western blot detection of the relative expression of circPCSK6‐167aa in PDX tissues from the high‐ and low‐circPCSK6 expression groups was performed (*n* = 9). D) Evaluation of the effect of gemcitabine on organoid growth after the upregulation of circPCSK6‐167aa in PDO‐6 cells was evaluated (*n* = 6). Scale bar: 200 µm. E) PDO‐6 organoids were subcutaneously transplanted into nude mice, which were then treated with gemcitabine (20 mg kg^−1^) to observe the PDOX size (*n* = 6). F) H&E staining and ki‐67 immunohistochemistry were performed on samples from the different PDOX‐6 groups (*n* = 6). Scale bar: 100 µm. Data in (A–C) were presented by two‐tailed Student's *t*‐test. Data in (D–F) were presented by two‐way ANOVA test. *p < 0.05; **p < 0.01; ***p < 0.001. Data are represented as mean ± SD.

To explore the translational potential of circPCSK6, we generated ICC PDX models in NKG mice (Figure S11A, Supporting Information). Employing the median expression levels of circPCSK6 as a demarcation, we segregated the PDX cohort into groups exhibiting high expression (PDX‐2, 4, and 9) and low expression (PDX‐3, 6, and 7). Subsequent subcutaneous engraftment into nude mice revealed significant differences in tumorigenic metrics, with the low‐expression circPCSK6 group manifesting significantly augmented tumor volume, weight, and ki‐67 positivity rates compared with its high‐expression counterpart (Figure 8A,B).

**Figure 9:**
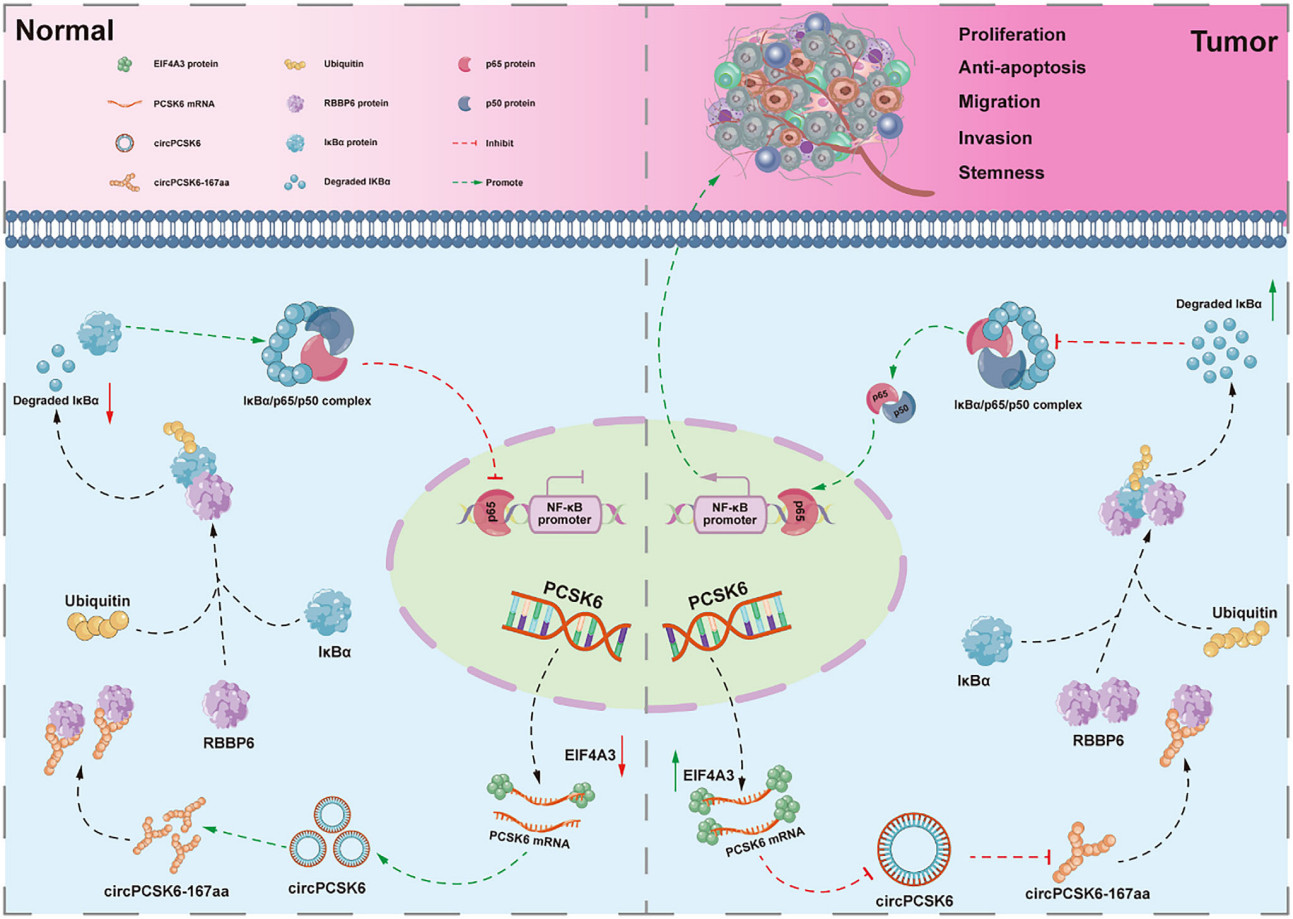
Mechanism of circPCSK6 inhibition of malignant behaviors in ICC. The tumor‐suppressive role of circPCSK6 in ICC cells is mediated through the encoding of a peptide, circPCSK6‐167aa, which interacts with the E3 ubiquitin ligase RBBP6. This interaction regulates the ubiquitination of IκBα within the NF‐κB signaling pathway, subsequently inhibiting the activation of this pathway. The suppression of NF‐κB pathway activation impacts tumor cell proliferation, migration, invasion, stemness characteristics, and hepatic‐lung metastasis. Additionally, compared with that in normal cells, the upstream regulator EIF4A3 in ICC cells promotes malignant behaviors by inhibiting the formation of circPCSK6, thereby exacerbating the malignancy of ICC cells.

Given that gemcitabine is a key component in ICC therapeutic strategies,^[^ ^23^ ^]^ we sought to determine the effect of circPCSK6‐167aa expression variability on gemcitabine resistance in ICC. Western blot analyses revealed that PDX‐6 and PDX‐9 as had the lowest and highest levels of circPCSK6‐167aa expression, respectively (Figure 8C). Using these models, we established ICC organoids (PDOs) and investigated their responsiveness to gemcitabine (Figure S11A, Supporting Information). The results from the CCK‐8 assay indicated that the high expression group displayed a lower half‐maximal inhibitory concentration (IC50) for gemcitabine than did the low circPCSK6‐167aa expression group (Figure S11B, Supporting Information). The subsequent overexpression of circPCSK6‐167aa in PDO‐6 cells resulted in increased sensitivity to gemcitabine (Figure 8D), whereas the knockdown of circPCSK6 in PDO‐9 cells increased gemcitabine resistance (Figure S11C, Supporting Information). Finally, the transfected PDOs were xenografted into nude mice to establish PDOX models (Figure S11D, Supporting Information). Once the tumor diameter reached 5 mm, PBS and gemcitabine (20 mg kg^−1^) were administered intraperitoneally every 3 days for 8 cycles (Figure S11A, Supporting Information). The low‐circPCSK6‐167aa group exhibited more pronounced inhibition of PDOX by gemcitabine when circPCSK6‐167aa was overexpressed (Figure 8E,F). Conversely, circPCSK6 downregulation in the high‐expression group corresponded with diminished therapeutic efficacy of gemcitabine (Figure S11E,F, Supporting Information). These findings collectively emphasize the pivotal role of circPCSK6‐167aa in modulating ICC resistance to gemcitabine and underscore its distinctive anticancer properties in ICC (**Figure** **9**).

## 3 Discussion

Intrahepatic cholangiocarcinoma is a prevalent form of liver cancer, with a notable increase in occurrence over recent years.^[^ ^24^ ^]^ Due to the lack of early diagnostic methods,^[^ ^25^ ^]^ ICC is often diagnosed at an advanced stage,^[^ ^26^ ^]^ and despite available treatments including surgery,^[^ ^4^, ^27^ ^]^ radiotherapy,^[^ ^28^ ^]^ and chemotherapy,^[^ ^29^ ^]^ the overall prognosis remains poor.^[^ ^5^, ^30^ ^]^ This highlights the urgency in identifying new biomarkers and therapeutic targets within this field. Circular RNAs are a unique class of non‐coding RNAs known for their high stability and diverse biological functions,^[^ ^31^ ^]^ such as regulating gene expression,^[^ ^32^ ^]^ acting as microRNA sponges,^[^ ^33^ ^]^ and influencing post‐transcriptional regulation.^[^ ^34^ ^]^ Although circRNAs play significant roles in various cancers,^[^ ^35^ ^]^ research into their functions and mechanisms in ICC is still limited.

Our investigation focused on circPCSK6 (hsa_circ_0037100), a circRNA that is markedly under expressed in ICC samples compared with normal liver tissue. This decrease is strongly correlated with adverse patient outcomes, suggesting that circPCSK6 is a potential tumor suppressor. Experimental approaches in both cell culture and animal models have substantiated the inhibitory effects of circPCSK6 on tumor growth, stem‐like properties, and metastatic capabilities.

Additionally, we investigated the possible post‐transcriptional mechanisms by which circPCSK6 might exert its influence. Specifically, it appears to contribute beyond its typical non‐coding functions, as it encodes a novel peptide, circPCSK6‐167aa, which was shown to notably downregulate the NF‐κB signaling pathway in ICC cells engineered to overexpress this peptide. Considering the integral role of the inflammatory milieu in ICC pathogenesis,^[^ ^36^ ^]^ with NF‐κB acting at the core of inflammation‐mediated cellular functions and chemoresistance, this finding is particularly compelling.^[^ ^38^ ^]^ Typically, the NF‐κB complex is held in check by IκBα,^[^ ^39^ ^]^ which obscures the complex's nuclear entry signal, thus inhibiting its nuclear transcriptional activities.^[^ ^40^ ^]^ This body of work not only highlights the potential of circPCSK6 as a critical element in the molecular landscape of ICC but also opens the door for novel therapeutic strategies targeting this pathway.

Our findings suggest that circPCSK6‐167aa orchestrates an unconventional method to control the stability of IκBα by influencing its ubiquitination, thereby stalling NF‐κB activation. Ubiquitination is pivotal for protein degradation and plays a central role in various biological phenomena,^[^ ^41^ ^]^ including tumor progression.^[^ ^42^ ^]^ Through protein interaction studies and ubiquitination assays, we revealed that circPCSK6‐167aa thwarts the proteasomal degradation pathway of IκBα, leading to the accumulation of non‐ubiquitinated IκBα in cells. This accumulation obstructs the movement of NF‐κB to the nucleus and its subsequent activity. Further experiments confirmed the interaction between circPCSK6‐167aa, RBBP6, and IκBα; specifically, site‐directed mutagenesis strategies were used to reveal that the binding of RBBP6 to IκBα depends on its RING domain, which is crucial for its E3 ligase activity. This competitive inhibition prevents RBBP6 from degrading IκBα, which safeguards IκBα from proteasomal breakdown. This nontypical regulatory mechanism of IκBα stability highlights the potential of circPCSK6‐167aa to indirectly modulate NF‐κB and proposes a unique “switch‐like” regulatory effect, suggesting that circPCSK6 may have broader applications in targeting multiple cancer‐related pathways simultaneously.

Additionally, our findings highlight the significant role of EIF4A3,^[^ ^43^ ^]^ as an RNA‐binding protein,^[^ ^44^ ^]^ which plays a significant role in regulating circPCSK6. Using prediction tools from the circInteractome platform, we initially suspected a strong interaction between EIF4A3 and the flanking sequences of circPCSK6. These findings suggest that EIF4A3 could regulate the production of circPCSK6 by affecting the splicing of these critical areas. Subsequent experiments confirmed that EIF4A3 indeed binds specifically with circPCSK6. This binding is crucial for the formation of the circular structure of circPCSK6, as its splicing efficiency directly affects the production rate of circular RNAs. Further experiments indicated that EIF4A3 suppresses the expression level of circPCSK6. These findings reveal how EIF4A3 indirectly affects the expression of circPCSK6 through its role in splicing regulation and further influences the functionality of the protein encoded by circPCSK6, circPCSK6‐167aa. The combined intervention of EIF4A3 and circPCSK6 highlights a promising avenue for comprehensive ICC treatment, where targeting EIF4A3 may enhance circPCSK6‐mediated anticarcinogenic effects, thus increasing treatment efficacy.

The identification of circPCSK6‐167aa redefines the conventional view of circular RNAs, which were previously categorized strictly as non‐coding elements, and underscores their potential as novel therapeutic targets in oncology. We expanded our investigation to examine the influence of circPCSK6‐167aa on drug resistance, employing PDX, PDO, and PDOX models. Our data show that PDX tumors expressing low levels of circPCSK6 proliferate more rapidly than those with elevated levels, confirming its tumor‐suppressive properties. PDO models replicate the tumor microenvironment,^[^ ^45^ ^]^ including intercellular interactions and the influence of the extracellular matrix,^[^ ^46^ ^]^ providing conditions closer to clinical reality for assessing the role of circPCSK6‐167aa. Observations from the PDO setup indicated that organoids with increased circPCSK6‐167aa levels exhibited stunted growth under gemcitabine treatment, highlighting its pivotal role in modulating chemotherapy responses. Similarly, the PDOX models, which were transplanted into live hosts, provided deeper insights into in vivo drug responses, showing that tumors enriched with circPCSK6‐167aa not only shrunk in size but also responded better to gemcitabine therapy. These models collectively suggest that enhancing circPCSK6‐167aa expression could increase chemosensitivity in ICC, offering new avenues for improving patient outcomes with existing chemotherapy regimens.

In addition, our study suggests that the regulatory role of circPCSK6 extends beyond the NF‐κB pathway, potentially through other ICC‐related molecular pathways, including those involved in tumor metabolism and extracellular matrix remodeling. Future research will focus on elucidating the broader influence of circPCSK6 in ICC and its interactions with diverse molecular targets, particularly how circPCSK6‐167aa might serve as a versatile modulator of multiple pathways, thus enhancing its value as an upstream “switch” molecule capable of orchestrating multifaceted anti‐cancer effects.

Overall, our future research will focus on delineating the interactions between circPCSK6‐167aa and various signaling molecules to clarify its specific contributions to ICC pathology. Furthermore, considering the regulatory effect of circPCSK6‐167aa on the NF‐κB pathway through the modulation of IκBα ubiquitination, it is imperative to dissect the intricate molecular mechanisms underlying this regulation. We also plan to further investigate the broader regulatory role of EIF4A3 and its potential combined impact with circPCSK6 in cancer treatment strategies. Advancing this line of inquiry could facilitate the development of targeted therapeutic options, potentially combining interventions on both circPCSK6 and EIF4A3 to maximize therapeutic impact. Such strategic interventions hold promise for enhancing the specificity and efficacy of ICC therapies, transforming the landscape of cancer treatment.

## 4 Experimental Section

### Clinical Samples

Tumor tissues and paired normal tissues were procured from 103 patients diagnosed with intrahepatic cholangiocarcinoma (ICC) at the 2nd Affiliated Hospital of Harbin Medical University. These tissues were procured through surgical resection, with comprehensive consent from each patient. Immediately after removal, the tissues were dissected under aseptic conditions and promptly cryopreserved in liquid nitrogen at −80 °C to preserve RNA and protein integrity. To prevent RNA degradation, RNase inhibitors were judiciously employed during sample processing. Patient data, including age, sex, clinical stage, and treatment history, were meticulously documented. All experimental protocols were approved by the relevant ethics committee (KY2021‐230).

### Cell Culture and Transfection

The intrahepatic cholangiocarcinoma cell lines RBE, HuCCT1, KMBC, and CCLP‐1 were cultured in DMEM (Gibco, New York, USA) or RPMI‐1640 (Gibco) supplemented with 10% fetal bovine serum (Invitrogen, Carlsbad, CA, USA) and 1% penicillin‐streptomycin (Gibco) under 37 °C with 5% CO_2_. Routine passaging was performed via 0.25% trypsin‐EDTA solution at 3‐day intervals. For the transfection experiments, Lipofectamine 2000 (Invitrogen) transfection reagent was used judiciously per the manufacturer's instructions. Predesigned shRNA vectors targeting circPCSK6 and control shRNA lentiviral vectors were combined with the transfection reagent and instilled into cells at approximately 60–70% confluence. Post‐transfection, the selective culture medium containing 2 µg mL^−1^ puromycin was replaced to screen for stable transfection until non‐transfected control cells were entirely eradicated, thereby ensuring optimal transfection efficiency. The detailed sequences are presented in Table S1 (Supporting Information).

### Quantitative Real‐Time PCR (qRT‐PCR)

qRT‐PCR assays were conducted using total RNA extracted from cells or tissue samples. Initially, total RNA was isolated via TRIzol reagent (Invitrogen), after which the RNA concentration and purity were assessed via a NanoDrop spectrophotometer (Bio‐Rad, Hercules, USA). A 260/280 ratio ranging between 1.8 and 2.1 was used to ensure that the RNA sample was suitable for subsequent analyses. Reverse transcription was performed with the PrimeScript RT Reagent Kit (Roche, Penzberg, Germany) according to the manufacturer's guidelines to transcribe 1 µg of total RNA into cDNA. Each qRT‐PCR reaction setup comprised 10 µL of SYBR Green PCR Master Mix, 1 µL of each primer (10 µm), 2 µL of cDNA template, and RNase‐free water to achieve a final volume of 20 µL. The cycling parameters included initial denaturation at 95 °C for 10 min, followed by 40 cycles of denaturation at 95 °C for 15 s and annealing/extension at 60 °C for 1 min. Each sample was subjected to triplicate technical replicates, with GAPDH serving as an internal reference gene for normalization. Relative expression levels were ascertained utilizing the 2^^−ΔΔCT^ method. The detailed primer sequences are expounded in the Table S1 (Supporting Information).

### Sanger Sequencing

To verify the reverse splicing site of circPCSK6, specific divergent primers were deployed to amplify circPCSK6 in ICC cells via PCR. The PCR reactions were conducted utilizing high‐fidelity polymerase to ensure precision in amplification. The resulting amplicons were separated and purified via agarose gel electrophoresis and sent to General Biol Company (Anhui, China) for Sanger sequencing, thereby enabling accurate identification of circPCSK6 splicing sites.

### RNase R assay

Total RNA was extracted from ICC cells and subjected to RNase R treatment to compare the stability of circPCSK6 and its parent gene PCSK6 mRNA both before and after treatment. Equal quantities of total RNA were partitioned into two aliquots, with one subjected to RNase R enzyme treatment and the other supplemented with an equivalent volume of RNase‐free control buffer. Following incubation at 37 °C for 20 min, the RNA was purified via an RNA purification kit (Qiagen, Hilden, Germany) for subsequent qRT‐PCR analysis.

### Subcellular Localization

Transfected cells were harvested, and subcellular fractions were isolated with a subcellular isolation kit (Thermo Fisher Scientific, Waltham, MA, USA) according to the manufacturer's instructions to separate the cells into cytoplasmic and nuclear fractions. RNA was then extracted from each fraction and analyzed via qRT‐PCR to determine circPCSK6 expression levels. GAPDH served as an internal reference for the cytoplasm, while U6 functioned as an internal reference for the nucleus. The relative expression of circPCSK6 in the cytoplasmic and nuclear compartments was computed to ascertain its predominant cellular localization.

### Fluorescence In Situ Hybridization (FISH)

FISH assays were conducted with custom fluorescently labeled probes (Ribobio, Guangzhou, China) tailored for circPCSK6. The cells were affixed on slides, fixed with 4% paraformaldehyde (Sigma, St. Louis, MO, USA) for 10 min at room temperature, and subsequently permeabilized with 0.5% Triton X‐100 (Sigma). The probes were hybridized overnight in a humid chamber at 37 °C. Following hybridization, the cells were washed several times with PBS and counterstained with a DAPI‐containing antifade solution before being covered with a coverslip. Photomicrography and visualization of circPCSK6 subcellular localization were performed via a fluorescence microscope (Leica, Wetzlar, Germany).

### EdU Assay

ICC cells were cultured in DMEM supplemented with 10% FBS until attaining 70% confluence, subsequently treated with 10 µm EdU Apollo567 In Vitro Imaging Kit (RiboBio), and further cultured for 2 h. Following treatment, cells were fixed and subjected to fluorescence labeling as per the kit instructions. Positively labeled cells were visualized under a fluorescence microscope, and the percentage of positively labeled cells was computed to assess cell proliferation activity.

### Tunel Assay

A Tunel (TdT‐mediated dUTP nick‐end labeling) assay was employed to evaluate apoptosis under baseline and drug‐induced conditions. Using the In Situ Cell Death Detection Kit, Fluorescein (Roche), fixed ICC cells were initially permeabilized with 0.1% Triton X‐100 solution in PBS for 10 min at room temperature. The cells were subsequently incubated with Tunel labeling mixture at 37 °C for 1 h in a humidified chamber. Following incubation, cells were washed with PBS and observed under a fluorescence microscope (model and manufacturer). For quantitative analysis, Tunel‐positive cells were counted in at least five random fields per sample, and the percentage of apoptotic cells was calculated to assess the degree of apoptosis under untreated and treated conditions.

### Transwell Assay

Transwell membranes (Corning, New York, USA) featuring 8.0 µm pores were used to evaluate ICC cell migration and invasion capabilities. For migration assessments, 5×10^4 cells suspended in medium devoid of FBS were added to the upper chamber, while medium containing 10% FBS was introduced into the lower chamber as a chemoattractant. For invasion assays, transwell membranes were precoated with Matrigel (BD Biosciences, San Jose, USA) to mimic the extracellular matrix barrier. After 24 h, non‐migrated or non‐invaded cells were eradicated via a cotton swab, whereas migrated or invaded cells in the lower chamber were immobilized with 4% paraformaldehyde and stained with 0.1% crystal violet. The number of migrated or invaded cells was subsequently determined via microscopy.

### Tumor Sphere Formation Assay

The tumor sphere formation assay was used to gauge the stem‐like properties of tumor cells. Single‐cell suspensions were dispensed into ultra‐low attachment 96‐well plates (Corning) at approximately 2000 cells per well, maintained in medium supplemented with B27 supplement, 20 ng mL^−1^ EGF, and 20 ng mL^−1^ bFGF (MedChemExpress, New Jersey, USA). Following 7–14 days of incubation, formed spheres were scrutinized and enumerated under an inverted microscope to assess cellular stemness.

### Western Blot

Protein extraction from ICC tissues or cells was performed via RIPA buffer (Thermo Fisher Scientific) supplemented with protease inhibitors (Sigma). The protein concentration was quantified using the BCA protein quantification kit (Beyotime, Beijing, China). Approximately 30 µg of protein per sample was subsequently subjected to SDS‐PAGE (10% gel) and subsequently transferred onto polyvinylidene fluoride membranes (PVDF, Millipore, Billerica, MA, USA). Membranes were blocked with 5% skim milk (Bio‐Rad) in TBS‐Tween 20 for 1 h, followed by overnight incubation with specific primary antibodies (e.g., E‐cadherin, N‐cadherin, Vimentin, SOX2, OCT4, NANOG) at 4 °C. The subsequent day, the membranes were incubated with the corresponding HRP‐conjugated secondary antibodies for 1 h. Signal visualization was performed via a chemiluminescent substrate (Thermo Fisher Scientific), and detection was performed via an imaging system (Bio‐Rad).

### Luciferase Reporter Gene Assay

The luciferase reporter gene assay facilitated the evaluation of IRES sequence activity. The target IRES sequence was cloned and inserted into the 5′ end of the luciferase gene via a luciferase reporter gene vector (pGL3‐Promoter Vector, Promega, Madison, USA). ICC cells were transfected with Lipofectamine 2000 after they reached 70% confluence in a 24‐well plate. A Renilla luciferase control reporter vector (pRL‐TK Renilla Luciferase Control Reporter Vector, Promega) was co‐transfected as an internal control. The cells were assayed via the Dual‐Luciferase Reporter Assay System (Promega) 48 h post‐transfection on a luminometer.

### Immunofluorescence (IF)

IF studies were performed to investigate the subcellular localization of 3×Flag circPCSK6‐167aa, IκBα, and RBBP6 in intrahepatic cholangiocarcinoma cells. The cells were seeded onto pre‐treated glass coverslips, fixed with 4% paraformaldehyde for 15 min after 24 h of culture, and permeabilized with 0.1% Triton X‐100 for 10 min to increase the permeability of the cell membrane. Nonspecific binding was confirmed by blocking with 1% BSA (Sigma) in PBS for 60 min. Overnight incubation with primary antibodies against Flag, IκBα, and RBBP6 was then performed (Abcam, Cambridge, USA), followed by subsequent washes with PBS and a 60‐min incubation with the corresponding fluorescent secondary antibodies (Alexa Fluor 488 and Alexa Fluor 594, Abcam) at room temperature in the dark. Nuclei were stained with DAPI (Thermo Fisher Scientific) for 5 min, washed with PBS, and cover slipped with antifade mounting medium (Beyotime), and observations were made via a Leica fluorescence microscope.

### RNA‐Seq

Total RNA extraction from ICC cells was carried out using TRIzol reagent, followed by purification with an RNA purification kit (manufacturer's directives). The RNA‐seq library construction and sequencing were performed by Novogene (Beijing, China). In brief, RNA quality and concentration were gauged with a NanoDrop spectrophotometer, with qualified samples (RNA integrity number ≥ 7) earmarked for library construction. The TruSeq RNA Sample Preparation Kit (Illumina, San Diego, USA) was used for library construction per the manufacturer's protocol. Subsequent library sequencing was performed on the Illumina HiSeq platform, yielding 150 bp paired‐end reads. The sequencing data were subjected to quality control, adapter trimming, and culling of low‐quality reads in preparation for subsequent transcriptome analysis.

### Co‐Immunoprecipitation (Co‐IP)

Total protein was extracted from ICC cells to facilitate the immunoprecipitation of target proteins via antibody‐specific interactions. Initially, protein A/G magnetic beads (Santa Cruz Biotechnology, Dallas, USA) pre‐cleared nonspecific binding within the samples. Specific antibodies (e.g., anti‐Flag or anti‐Myc) were subsequently introduced into the cell lysate, followed by overnight incubation at 4 °C. On the following day, protein A/G magnetic beads were used to capture the antibody‐protein complexes, which were subjected to rotation at 4 °C for 2 h. After three washes with PBS, the proteins were eluted by boiling the mixture in 2× SDS sample buffer at 95 °C for 5 min, after which they were primed for downstream western blot analysis.

### Silver Staining

SDS‐PAGE‐separated proteins were subjected to silver staining for protein band detection. A silver staining kit (Beyotime) was used according to the manufacturer's instructions. Gel immersion in fixing solution for 30 min commenced the procedure, followed by multiple SDS washes to eliminate residual SDS. The samples were subsequently immersed in staining solution until discernible bands emerged. Culmination transpired with the termination of the staining reaction via stop solution, followed by washing with water to expunge background noise.

### Immunoprecipitation‐Mass Spectrometry (IP‐MS)

Following extraction and enrichment of target protein complexes from ICC cells using Co‐IP, proteins were enzymatically digested into peptides. The purified peptides were analyzed via liquid chromatography‐tandem mass spectrometry (LC‐MS/MS) which was supported by Jingjie PTM BioLabs (Hangzhou, China). Chromatographic separation of peptides, coupled with electrospray ionization and injection into the mass spectrometer, facilitated MS and MS/MS analysis. Software such as MaxQuant or Proteome Discoverer undertook processing of raw mass spectrometry data for protein identification and quantification, thereby unveiling potential interaction partners in Co‐IP.

### Ni‐NTA Pull Down

This method was utilized to verify the interaction between the His‐tagged IκBα protein and Flag‐tagged circPCSK6‐167aa or Myc‐tagged RBBP6. The initial transfection of 293T cells with the His‐tagged IκBα expression vector was followed by protein extraction and subsequent incubation with Ni‐NTA beads (Qiagen) to facilitate the binding of His‐tagged IκBα with the beads. The beads were washed with wash buffer containing 20 mm imidazole to eliminate nonspecifically bound proteins. Subsequently, the lysates from 293T cells expressing Flag‐tagged circPCSK6‐167aa or Myc‐tagged RBBP6 were incubated for 2 h to promote interactions between the target proteins and His‐tagged IκBα. After washing with imidazole‐containing buffer, the bound proteins were eluted with elution buffer containing 250 mm imidazole, and the samples were subjected to SDS‐PAGE and western blot analysis.

### RNA Immunoprecipitation (RIP)

To investigate the interaction between circPCSK6 and EIF4A3, RIP experiments were performed. First, lysed intrahepatic cholangiocarcinoma cells were treated with RIP buffer containing specific constituents. The RNA‐protein complexes in the lysate were subjected to immunoprecipitation via antibodies against EIF4A3 (Cell Signaling Technology, Danvers, USA) and A/G magnetic beads. After overnight incubation, the beads were collected and washed with low‐salt and high‐salt RIP wash buffers to prevent nonspecific binding. Finally, RNA was extracted from the precipitate, followed by reverse transcription and qRT‐PCR analysis, facilitating the assessment of the efficiency of EIF4A3 binding to circPCSK6.

### ICC Mouse Model, In Vivo Mouse Subcutaneous Tumor and Metastasis Models

The establishment of the ICC mouse model involved the rapid tail vein injection of a mixed plasmid mixture into 6‐week‐old C57BL/6 mice (Cyagen Biosciences, Jiangsu, China). The plasmids, comprising 15 µg pT3‐EF1α‐myr‐AKT1, 15 µg pT3‐EF1α‐NICD, and 3 µg pCMV‐SB, were diluted to a total volume of 2 mL of sterile saline and passed through a 0.22‐µm sterile filter to ensure sterility. The solution was injected within 5 s to ensure efficient delivery into the systemic circulation. Four weeks post‐injection, the mice were euthanized, and tissues were harvested for further analysis. Mouse subcutaneous tumor models were established via the suspension of liver intrahepatic cholangiocarcinoma cell lines in sterile saline and subsequently injected into the right subcutaneous area of nude mice (Cyagen Biosciences). Tumor growth was monitored regularly, and tumor volume was calculated. Metastasis models (liver and lung) were generated via intrasplenic and tail vein injections of cells into mice, respectively. Subsequently, liver and lung tissues were collected, and pathological changes in the liver and lung were observed through follow‐up tissue sections. The Ethics Committee of the 2nd Affiliated Hospital of Harbin Medical University approved all animal experiments (SYDW2021‐052).

### Paraffin Immunohistochemistry (IHC) and Hematoxylin‐Eosin (H&E) Staining

Tumor and organ tissue samples were fixed in 10% neutral buffered formalin for 24 h, followed by dehydration, clearing, and embedding in paraffin blocks. Microtome‐guided cutting of consecutive tissue sections (4 µm thickness) culminated in slide placement. Deparaffinization in xylene and dehydration in ethanol‐water solutions preceded H&E staining, which involved nuclear staining with hematoxylin (Solarbio, Beijing, China) for 5 min, acid alcohol differentiation, and eosin (Solarbio) counterstaining for 2 min. After dehydration, clearing, and cover slipping with neutral resin, immunohistochemical analysis was subsequently performed with specific antibodies, such as anti‐ki‐67 antibody (Abcam). Antigen retrieval, which typically employs citrate buffer for high‐pressure heat retrieval, preceded overnight incubation with primary antibodies. Detection was performed via a biotinylated secondary antibody system, culminating in DAB staining and nuclear counterstaining. Microscopic evaluation of the staining results was subsequently performed, accompanied by image capture.

### Patient‐Derived Xenograft (PDX) and Organoid (PDO) Models

PDX models entailed subcutaneous implantation of fresh tumor tissue samples into c‐NKG (Cyagen Biosciences) and nude mice (Cyagen Biosciences), following initial engraftment and passage for two generations. Successful engraftment led to subsequent transplantation into nude mice to establish PDX models, which were categorized into high and low‐expression groups on the basis of circPCSK6 median expression. PDO models originate from selected tumor tissues, were processed to isolate single cells or small clusters, and were subsequently cultured in Matrigel to support 3D growth and organoid formation. PDOX models were established by implanting in vitro‐cultured PDOs into immunodeficient mice, with subsequent monitoring of tumor growth inhibition and biological changes via intraperitoneal injection of gemcitabine (MedChemExpress).

### Half‐Maximal Inhibitory Concentration Detection of PDOs

To determine the half‐maximal inhibitory concentration (IC50) of gemcitabine for PDOs established from intrahepatic cholangiocarcinoma PDXs, PDOs were cultured in 96‐well plates and treated with varying concentrations of gemcitabine ranging from 0.1 to 100 µm to establish dose‐response curves, and the cultures were incubated for an additional 72 h. Cell viability was assessed via a Cell Counting Kit‐8 (CCK‐8; Dojindo, Kumamoto, Japan). Ten microliters of CCK‐8 solution was added to each well, followed by incubation at 37 °C for 2–4 h, and the absorbance was measured at 450 nm via a microplate reader. The IC50 values were calculated by fitting the data to a four‐parameter logistic model via nonlinear regression analysis.

### Bioinformatics Analysis

The GSE181523 gene expression dataset was used to identify differentially expressed genes (DEGs) between the ICC and control groups via bioinformatics methods. Data preprocessing included quality control, background correction, and normalization. Differential expression analysis was conducted via the limma package in R software, and the results were visually represented via volcano plots and heatmaps. The functional and pathway enrichment analyses of the DEGs involved Gene Ontology (GO) analysis and Kyoto Encyclopedia of Genes and Genomes (KEGG) analysis, which were used to elucidate biological processes and signaling pathways.

### Statistical Analysis

Statistical analyses were conducted via SPSS software (IBM SPSS, New York, USA), with the data tested for normality to determine appropriate testing methods. For normally distributed data, the independent samples *t*‐test or ANOVA was applied, whereas the Mann–Whitney U test or Kruskal–Wallis test was used for non‐normally distributed data. Survival analysis was performed via the Kaplan–Meier method and Cox proportional hazards model. Graphical representations were generated via GraphPad Prism 8.0 (GraphPad Software, San Diego, CA, USA) to visualize the data distribution and research outcomes. *p‐*values less than 0.05 were considered statistically significant.

## Conflict of Interest

The authors declare no conflict of interest.

## Author Contributions

C.G., J.G., and X.Z. contributed equally to this work. C.G., J.G., and X.Z. were responsible for the study of concept and design. C.G.J., G.W.S., and Y.H. performed the main experiments and analyzed the data. Y.G., Z.X., C.Y., and S.B. collected the clinical samples and collected the clinical data. P.K. and X.X. were responsible for study supervision. X.J. and X.Z. provided the experimental resources. C.G. and X.Z. wrote and modified the manuscript. P.K., X.X., and X.Z. supervised the manuscript. All authors have read and approved the final manuscript.

## Supporting information



Supporting Information

## Data Availability

The data that support the findings of this study are available from the corresponding author upon reasonable request.
