# Supplementary material for: A Novel 167‐Amino Acid Protein Encoded by CircPCSK6 Inhibits Intrahepatic Cholangiocarcinoma Progression via IKBα Ubiquitination
Source: Adv Sci (Weinh). 2025 Jan 21;12(10):2409173. doi: 10.1002/advs.202409173 (PMC11904980; doi:10.1002/advs.202409173)
Supplement: Supplementary file 1 — Supporting Information [file ADVS-12-2409173-s001.docx]

**Table S1 Primer sequences for qRT-PCR and siRNAs sequences.**

| Nucleic acids | Sequences |
| --- | --- |
| GAPDH | F: 5’-GAAGGTGAAGGTCGGAGT-3’ |
|  | R: 5’-GAAGATGGTGATGGGATTTC-3’ |
| U6 | F: 5’-GCTTCGGCAGCACATATACTAAA-3’ |
|  | R: 5’-CGCTTCACGAATTTGCGTGTC-3’ |
| circPCSK6 | F: 5’-CCTTGGCTCTAGAAGCAAAG-3’ |
|  | R: 5’-CACAACGAGTGCCGTGTTTA-3’ |
| PCSK6 | F: 5’-TGATTCCTACGCCAGCTACGAC-3’ |
|  | R: 5’-TTGTACGCTATGCCCACGATG-3’ |
| EIF4A3 | F: 5’-TGGCTCCCACAAGAGAGTT-3’ |
|  | R: 5’-GCACTGGACATTCATGTAGTCA-3’ |
| sh-NC | F: 5’-UUCUCCGAACGUGUCACGUTT-3’ |
|  | R: 5’-ACGUGACACGUUCGGAGAATT-3’ |
| sh-circ-1 | F: 5’-GAAGCAAAGAUUCCUACGCCATT-3’ |
|  | R: 5’-UGGCGUAGGAAUCUUUGCUUCTT-3’ |
| sh-circ-2 | F: 5’-CUCUAGAAGCAAAGAUUCCUATT-3’ |
|  | R: 5’-UAGGAAUCUUUGCUUCUAGAGTT-3’ |
| sh-circ-3 | F: 5’-GGCUCUAGAAGCAAAGAUUCCTT-3’ |
|  | R: 5’-GGAAUCUUUGCUUCGAGAGCCTT-3’ |
| si-PCSK6 | 5’-TGGAAGATTACTACCATTTTTAT-3’ |
| si-EIF4A3 | 5’-CUCUCGGUGACUACAUGAATT-3’ |

**
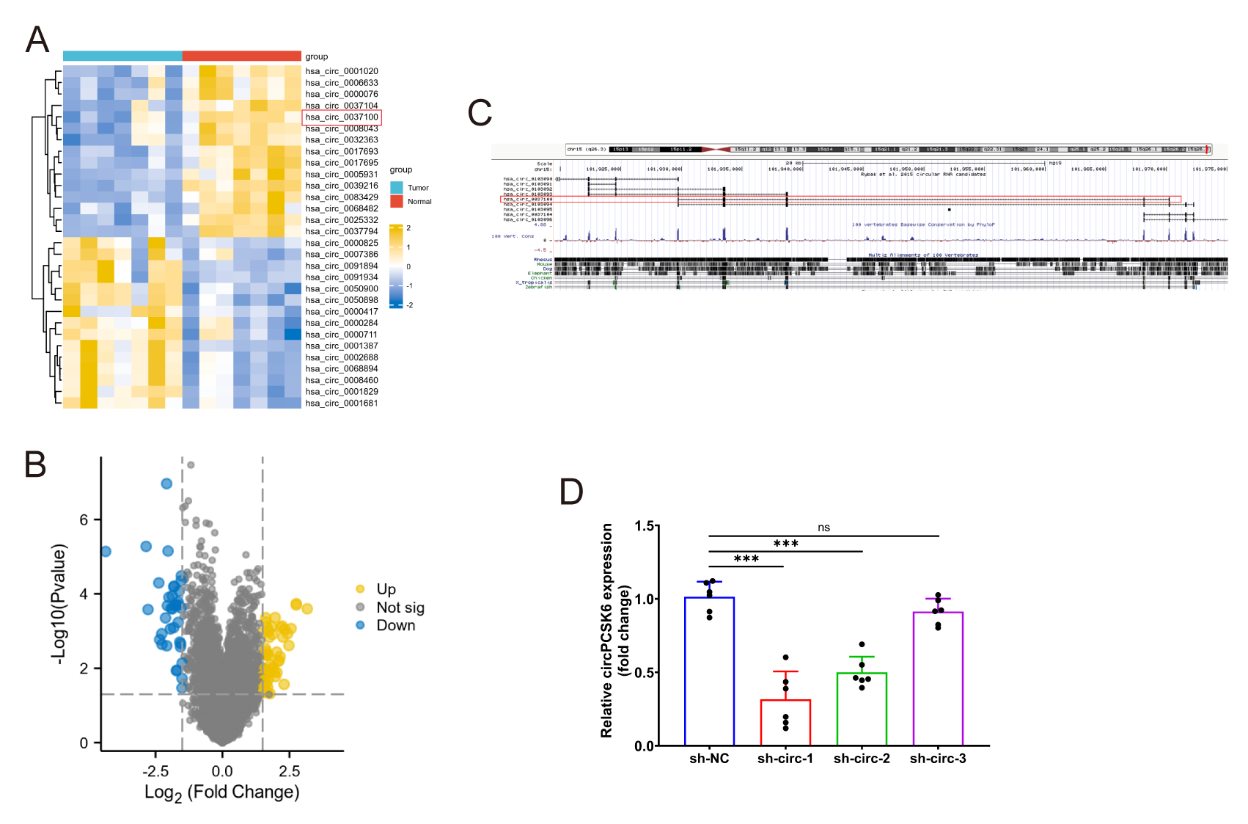
**

**Figure S1.** circPCSK6 expression in ICC. A, B) Heatmap and volcano plot of high and low circRNA expression in the GSE181523 database. C) Chart showing that circPCSK6 originates from the back-splicing of exons 6 to 9 of the linear form of PCSK6 mRNA. D) H&E staining of liver tissue in wild mice and primary ICC mice. E) Identification of the knockdown efficiency of three circPCSK6 siRNAs (n = 6). Data in (E) was presented by one-way ANOVA test. ****p* < 0.001. Data are represented as mean±SD.


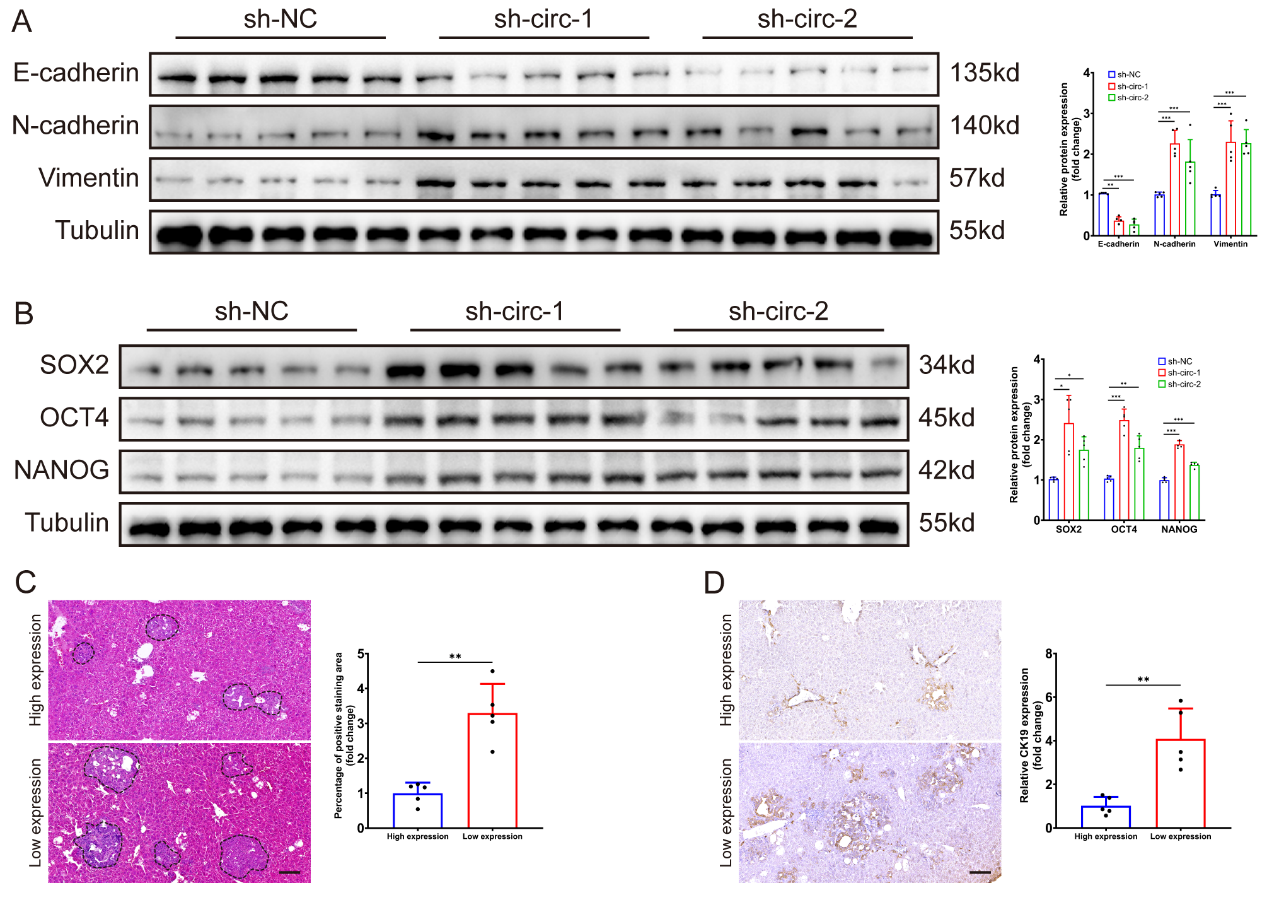


**Figure S2.** Low circPCSK6 expression promotes the progression of xenograft tumors and primary ICC in mice. A, B) Western blot analysis focusing on the changes in the expression of EMT and tumor stem cell-related markers (n = 5). C) H&E staining of primary ICC tissues in circPCSK6 high and low expression groups (n = 5). D) CK19 staining of mice primary ICC tissues in each group (n = 5). Scale bar: 100 μm. Data in (A, B) were presented by two-way ANOVA test. Data in (C, D) were presented by one-way ANOVA test. **p* < 0.05; ***p* < 0.01; ****p* < 0.001. Data are represented as mean±SD.


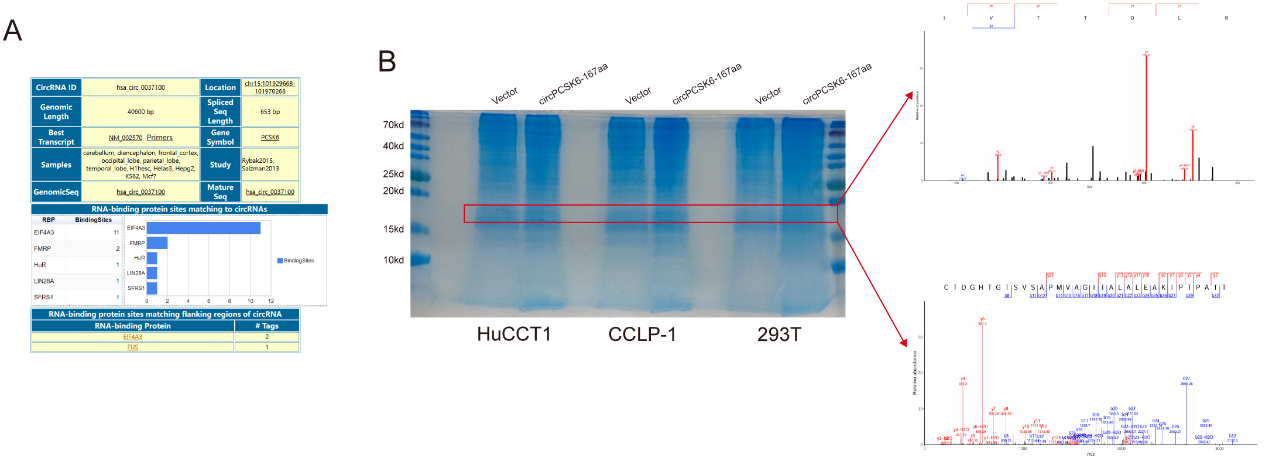


**Figure S3.** Identification of the novel protein circPCSK6-167aa. A) circInteractome prediction of potential RBP molecules binding to circPCSK6. B) Left panel: Coomassie blue staining images of exogenously expressed circPCSK6-167aa in HuCCT1, CCLP-1, and 293T cells; Right panel: specific peptides of circPCSK6-167aa detected by mass spectrometry.


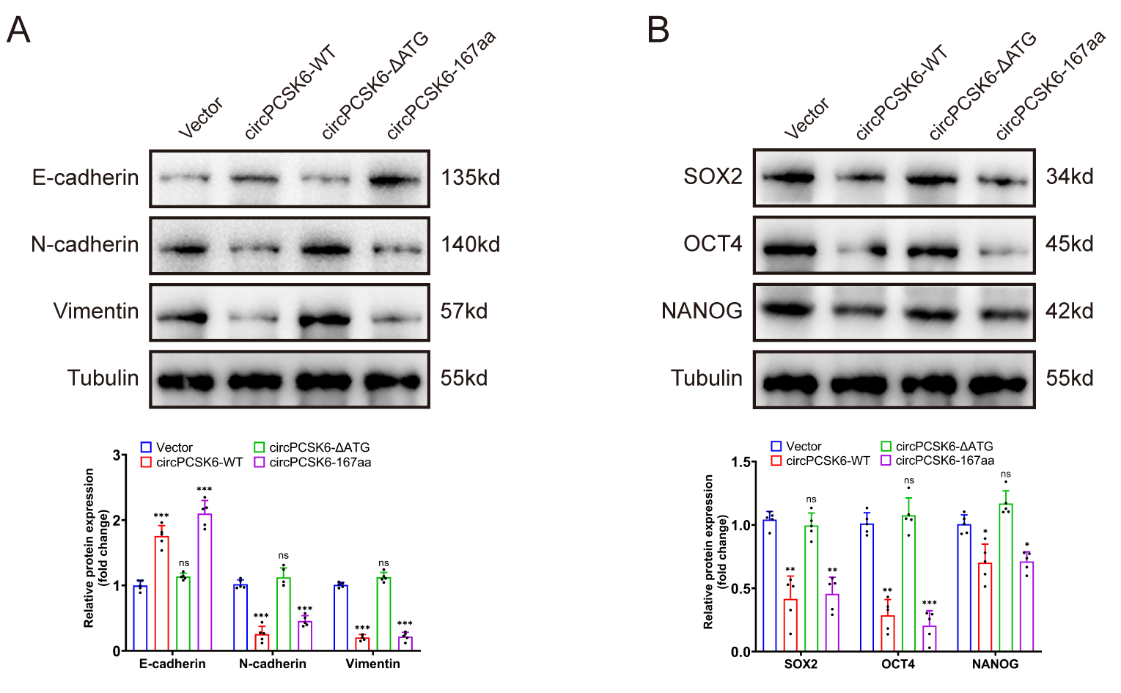


**Figure S4.** Expression of subcutaneous tumor EMT and tumor stem markers. A) Western blot analysis focusing on the changes in the expression of EMT markers (n = 5). B) Western blot analysis focusing on the changes in the expression of tumor stem cell-related markers (n = 5). Data in (A, B) were presented by two-way ANOVA test. **p* < 0.05; ***p* < 0.01; ****p* < 0.001. Data are represented as mean±SD.


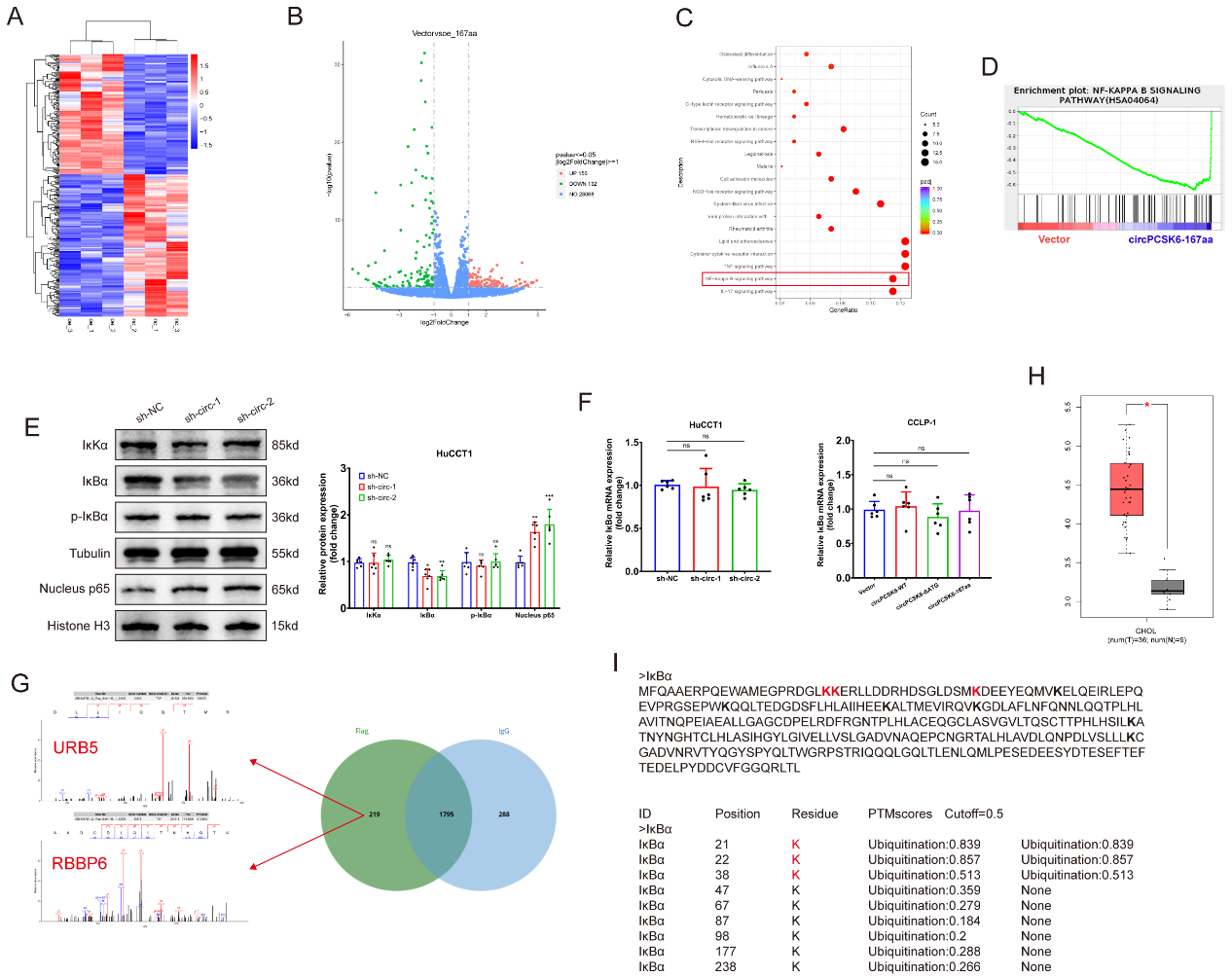


**Figure S5.** Regulation of NF-κB signaling pathway by circPCSK6-167aa in CCLP-1. A, B) Heatmap and volcano plot of differentially expressed genes identified by RNA-seq after overexpression of circPCSK6-167aa. C, D) Enrichment of differentially expressed genes in KEGG signaling pathways and GSEA analysis of the NF-κB signaling pathway. E) Expression of NF-κB signaling pathway-related proteins after silencing circPCSK6 (n = 6). F) qRT-PCR detection of relative expression of IKBα after silencing or overexpressing circPCSK6 (n = 6). G) IP-MS identification of E3 ligases interacting with circPCSK6-167aa, including UBR5 and RBBP6. H) GEPIA analysis showing abnormal high expression of RBBP6 in ICC. I) Prediction of possible ubiquitination sites on IKBα (K21, K22, K38) using MusiteDeep and UbPred. Data in (E) was presented by two-way ANOVA test. Data in (F) was presented by one-way ANOVA test. **p* < 0.05; ***p* < 0.01; ****p* < 0.001. Data are represented as mean±SD.


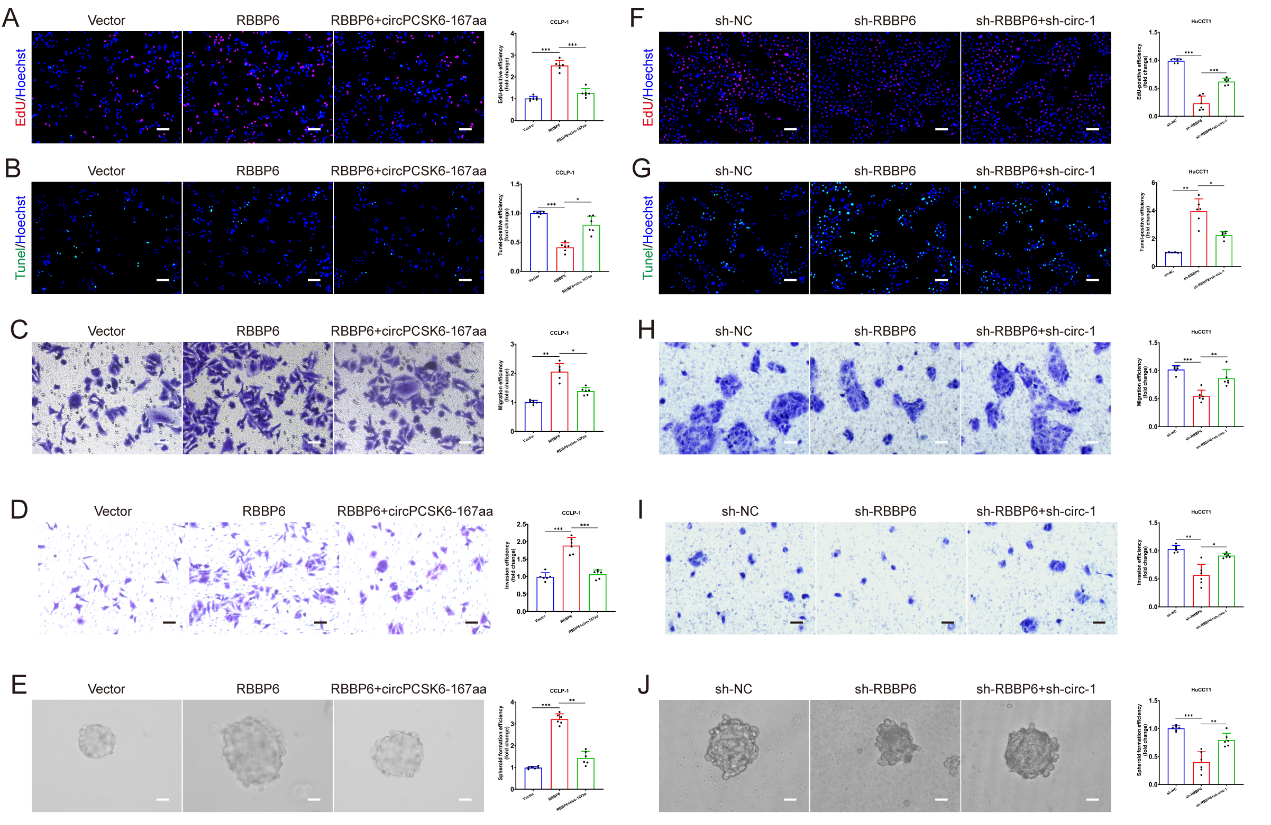


**Figure S6.** Regulatory effect of circPCSK6-167aa and RBBP6 on malignant biological behavior of ICC. A, B) EdU and Tunel assays to detect the proliferation and apoptosis abilities of CCLP-1 after transfection with empty vector, RBBP6, and co-transfection with RBBP6 and circPCSK6-167aa (n = 6). Scale bar: 100 μm. C, D) Transwell assays showing that the promotion of migration and invasion in CCLP-1 cells by upregulation of RBBP6 is reversed by circPCSK6-167aa (n = 6). Scale bar: 50 μm. E) Sphere formation assays to detect the rescue effect of circPCSK6-167aa on RBBP6-mediated promotion of tumor stemness (n = 6). Scale bar: 100 μm. F, G) EdU and Tunel assays to detect the proliferation and apoptosis abilities of CCLP-1 after transfection with sh-NC, sh-RBBP6, and co-transfection with sh-RBBP6 and sh-circPCSK6 (n = 6). Scale bar: 100 μm. H, I) Transwell assays showed that the inhibitory effect of down-regulation of RBBP6 on the migration and invasion of HuCCT1 cells was reversed by down-regulation of circPCSK6 (n = 6). Scale bar: 50 μm. J) Sphere formation assays to detect the rescue effect of circPCSK6 on RBBP6-mediated inhibition of tumor stemness (n = 6). Scale bar: 100 μm. Data in (A-J) were presented by one-way ANOVA test. **p* < 0.05; ***p* < 0.01; ****p* < 0.001. Data are represented as mean±SD.


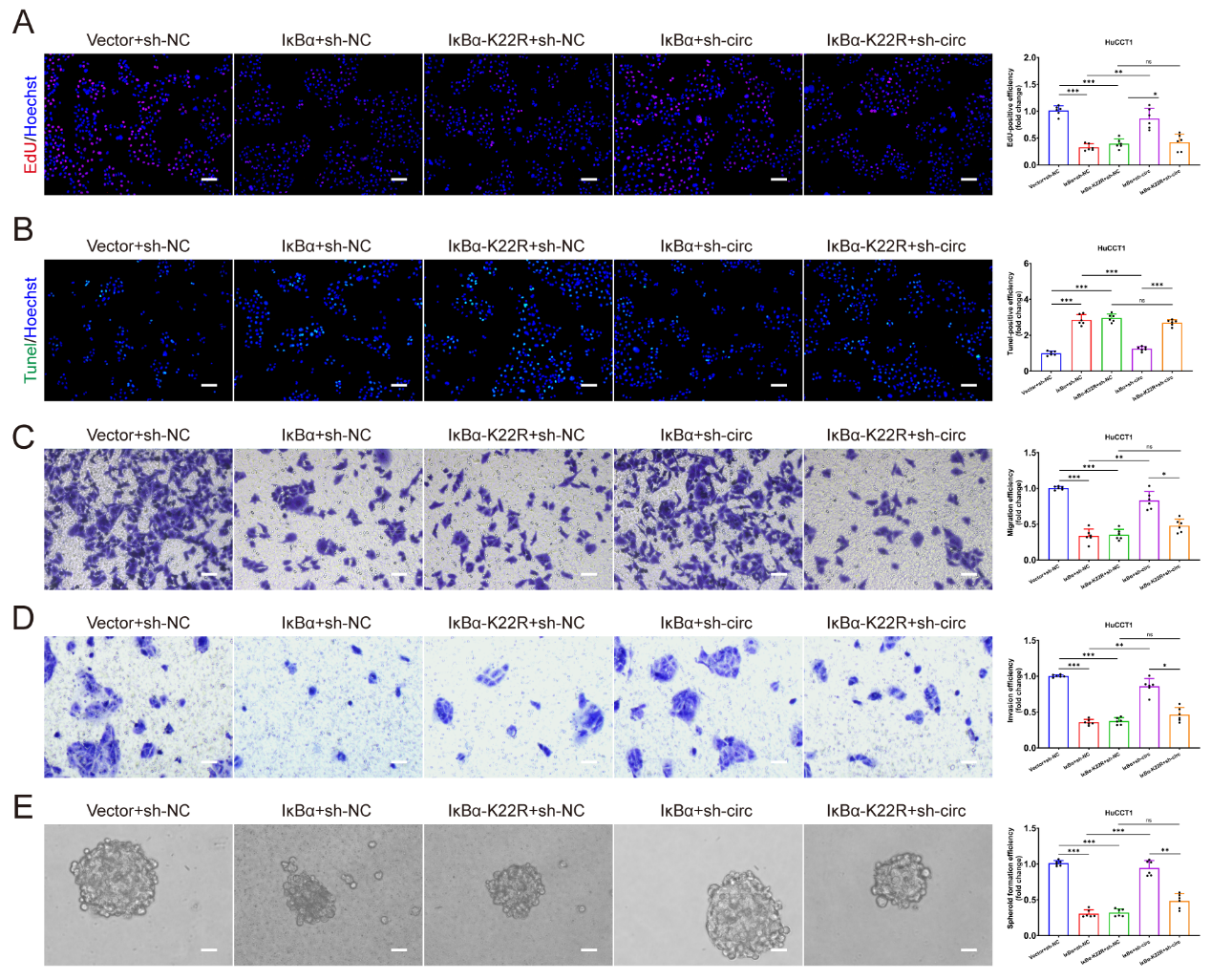


**Figure S7.** Downregulation of circPCSK6 can restore the inhibition of HuCCT1 progression caused by overexpression of IKBα, but not the IKBα K22 mutation (IKBα-K22R). A, B) EdU and Tunel assays show that the regulation of proliferation and apoptosis of HuCCT1 by IKBα can be reversed by knockdown of circPCSK6, but not by IKBα-K22R (n = 6). Scale bar: 100 μm. C, D) Transwell assays to detect the migration and invasion abilities of HuCCT1 cells after transfection with empty vector, IKBα, IKBα-K22R, IKBα+sh-circ, and IKBα-K22R+sh-circ (n = 6). Scale bar: 50 μm. E Sphere formation assays to detect the rescue effect of silencing circPCSK6 on the inhibition of tumor stemness by wild-type and mutant IKBα (n = 6). Scale bar: 100 μm. Data in (A-E) were presented by one-way ANOVA test. **p* < 0.05; ***p* < 0.01; ****p* < 0.001. Data are represented as mean±SD.


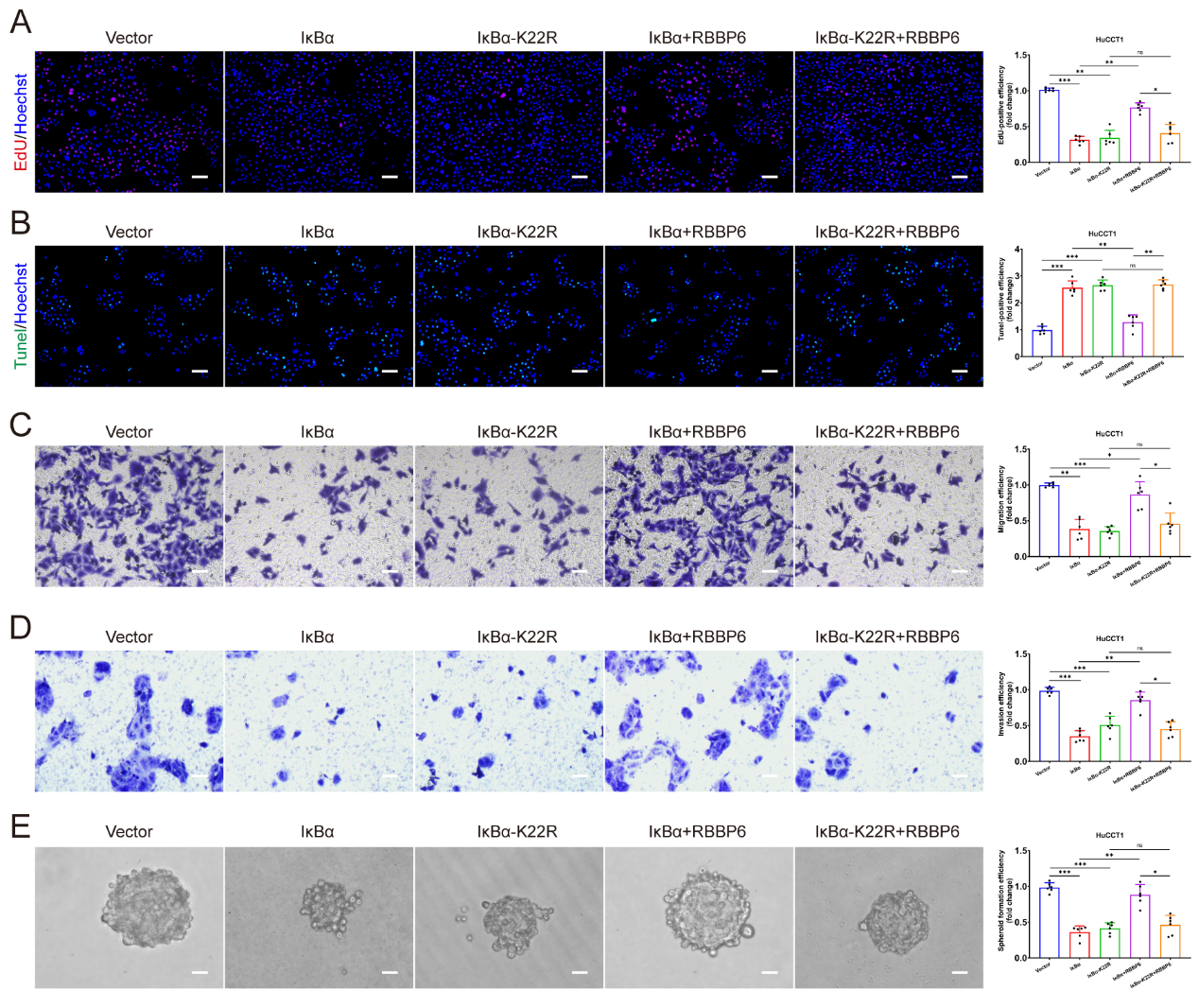


**Figure S8.** The inhibitory effect of IKBα overexpression on ICC progression can be rescued by overexpression of RBBP6, but the inhibitory effect of IKBα-K22R on the tumor cannot be reversed by RBBP6. A, B) EdU and Tunel assays to detect the proliferation and apoptosis abilities of HuCCT1 cells after transfection with empty vector, IKBα, IKBα-K22R, IKBα+RBBP6, and IKBα-K22R+RBBP6 (n = 6). Scale bar: 100 μm. C, D) Transwell assays show that the inhibitory effect of IKBα on the migration and invasion of HuCCT1 is reversed by upregulation of RBBP6, but IKBα-K22R is not affected by RBBP6 (n = 6). Scale bar: 50 μm. E Sphere formation assays to detect the rescue effect of overexpression of RBBP6 on the inhibition of tumor stemness by wild-type and mutant IKBα (n = 6). Scale bar: 100 μm. Data in (A-E) were presented by one-way ANOVA test. **p* < 0.05; ***p* < 0.01; ****p* < 0.001. Data are represented as mean±SD.


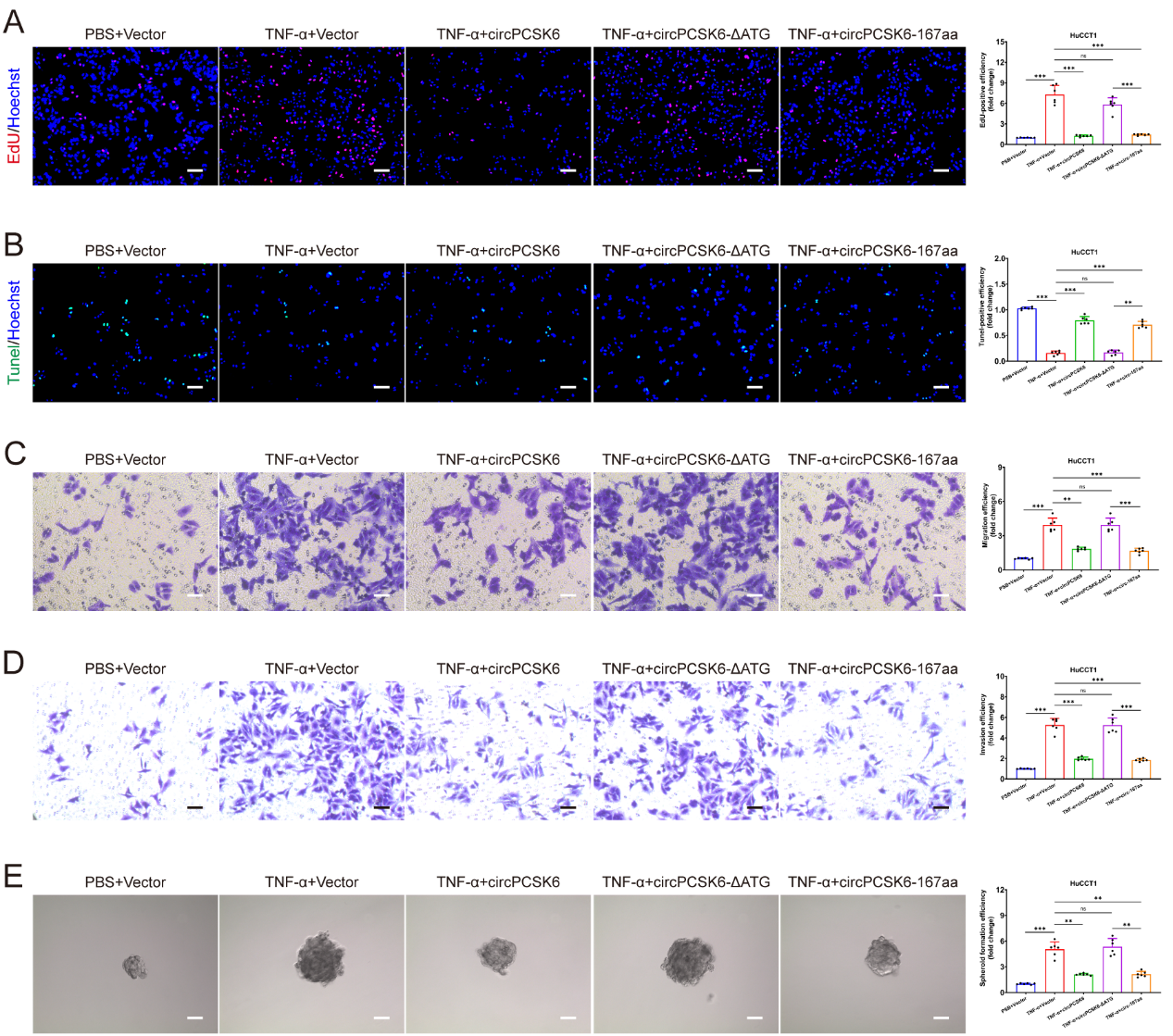


**Figure S9.** The promoting effect of TNF-α on ICC progression can be restored by overexpression of circPCSK6 and circPCSK6-167aa. A, B) Proliferation and apoptosis abilities of HuCCT1 detected by EdU and Tunel assays, respectively (n = 6). Scale bar: 100 μm. C, D) Transwell assays showing that both circPCSK6 and circPCSK6-167aa can reverse the promotion of migration and invasion of HuCCT1 cells induced by TNF-α, while circPCSK6-ΔATG cannot (n = 6). Scale bar: 50 μm. E) Sphere formation assays used to detect the ability of the three circPCSK6 overexpression vectors to rescue the tumor stemness promoted by TNF-α (n = 6). Scale bar: 100 μm. Data in (A-E) were presented by one-way ANOVA test. **p* < 0.05; ***p* < 0.01; ****p* < 0.001. Data are represented as mean±SD.

**
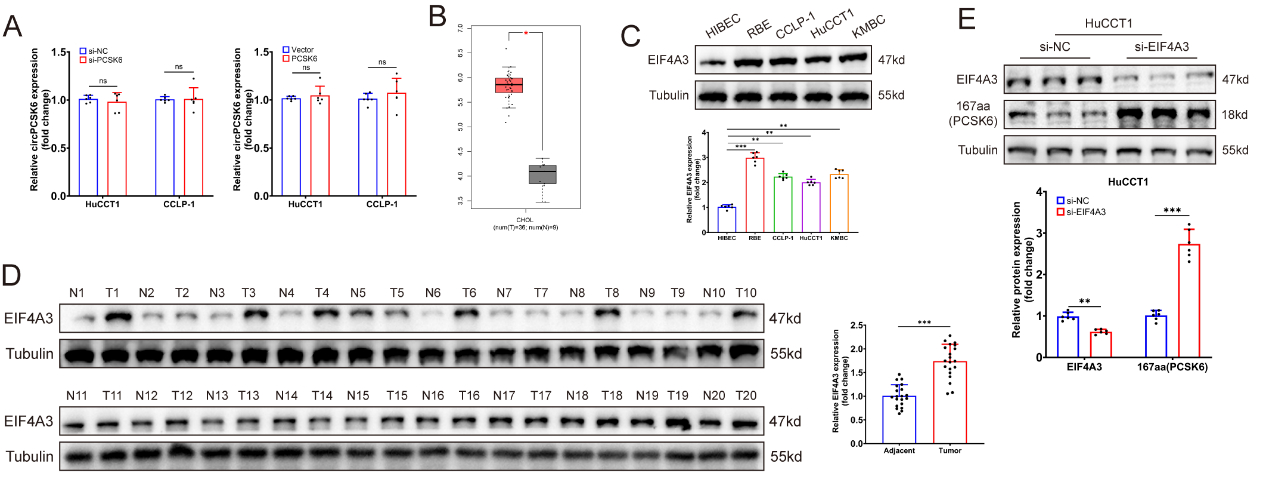
**

**Figure S 10.** Expression of EIF4A3 in ICC. A) qRT-PCR detection of the regulation of circPCSK6 expression after silencing or overexpressing the parental gene PCSK6 (n = 6). B) GEPIA analysis showing abnormal high expression of EIF4A3 in ICC. C, D) Western blot detection of the relative expression of EIF4A3 in ICC cells (n = 6) and tissues (n = 20). E) Western blot is used to detect the efficiency of si-EIF4A3 transfection into HuCCT1 cells and the expression of circPCSK6-167aa (n = 6). Data in (A, E) were presented by two-way ANOVA test. Data in (C) was presented by one-way ANOVA test. Data in (D) was presented by two-tailed Student's *t*-test. ***p* < 0.01; ****p* < 0.001. Data are represented as mean±SD.


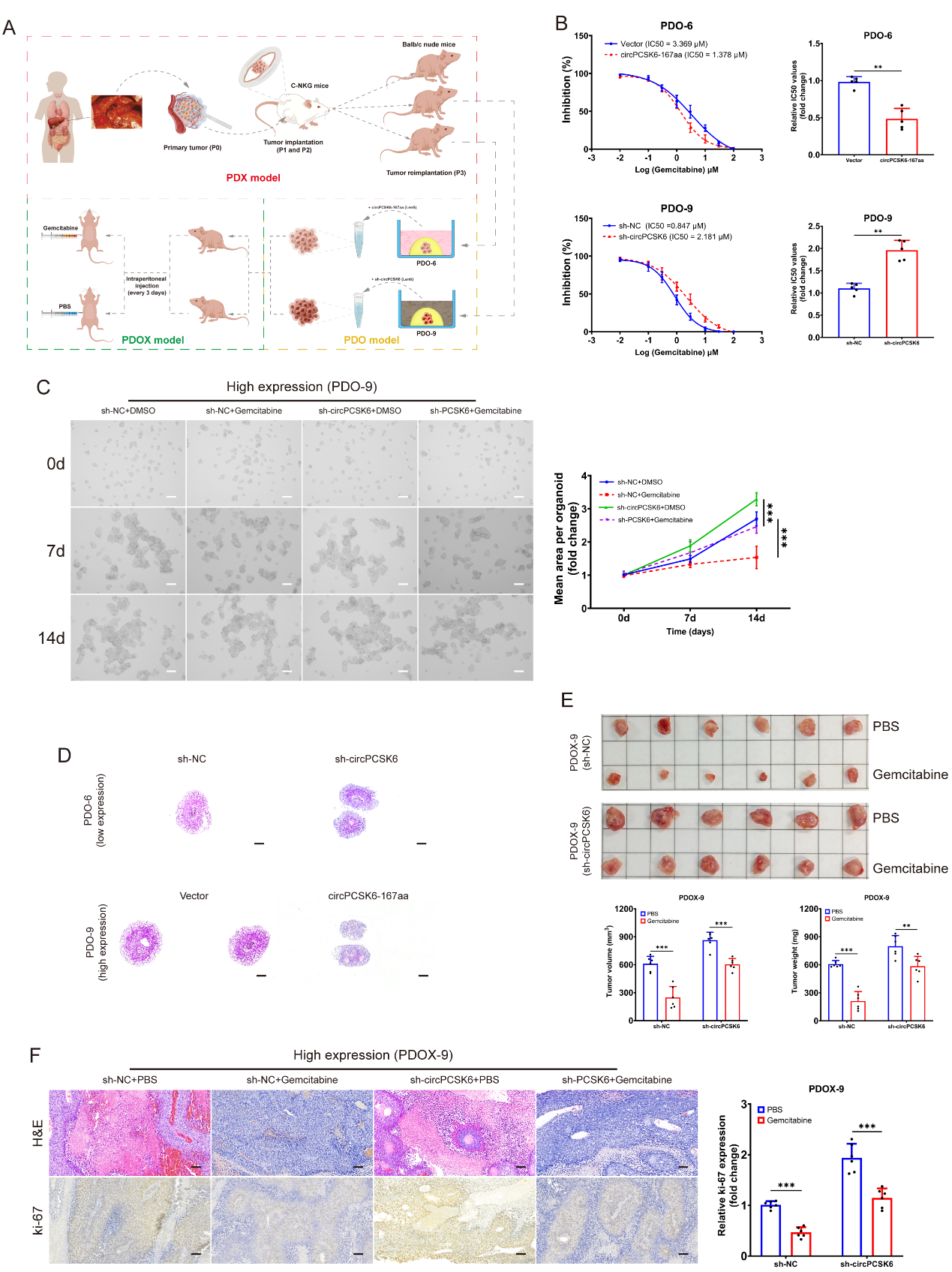


**Figure S 11.** circPCSK6 as a potential therapeutic target for ICC patients. A) Flowchart of the construction of PDX, PDO, and PDOX models. B) Determination of gemcitabine IC50 for PDO-6 and PDO-9 by constructing dose-response curves, and the effect of circPCSK6 on IC50 of these organoids (n = 6). C) Evaluation of the effect of gemcitabine on organoid growth after silencing circPCSK6 in PDO-9 (n = 6). Scale bar: 200 μm. D) H&E staining of organoids PDO-6 and PDO-9 before constructing PDOX models. Scale bar: 200 μm. E) Subcutaneous construction of PDOX-9 in nude mice and observation of PDOX weight and volume in different groups (n = 6). F) H&E staining and ki-67 IHC of PDOX-9 in different groups (n = 6). Scale bar: 100 μm. Data in (B) was presented by two-tailed Student's *t*-test and Nonlinear regression. Data in (C, E, F) were presented by two-way ANOVA test. ***p* < 0.01; ****p* < 0.001. Data are represented as mean±SD.
